# Efficient generation of male germ-like cells derived during co-culturing of adipose-derived mesenchymal stem cells with Sertoli cells under retinoic acid and testosterone induction

**DOI:** 10.1186/s13287-019-1181-5

**Published:** 2019-03-13

**Authors:** Yanxia Luo, Lili Xie, Ali Mohsin, Waqas Ahmed, Chenze Xu, Yan Peng, Haifeng Hang, Yingping Zhuang, Ju Chu, Meijin Guo

**Affiliations:** State Key Laboratory of Bioreactor Engineering, East China University of Science and Technology, 130 Meilong Rd, Shanghai, 200237 China

**Keywords:** Adipose-derived mesenchymal stem cells, Germ cell, Sertoli cell, Signaling pathways, Retinoic acid, Testosterone, Synergistic effect

## Abstract

**Background:**

Adipose-derived mesenchymal stem cells (ADMSCs) are considered an efficient and important candidate for male infertility treatment because they contain pluripotent stem cells, which can differentiate into all cells from three germ layers. However, the efficient generation of male germ-like cell (MGLCs) is one of the key issues, and little is known about the mechanisms underlying generation of MGLCs. Herein, we attempt to improve the efficient generation of MGLCs derived during co-culturing of rat ADMSCs with SCs under retinoic acid (RA) and testosterone (T) treatment.

**Methods:**

ADMSCs isolated from male SD rat were induced into generation of MGLCs by using respective methods in vitro. Transwell insert system was used for co-culturing. Busulfan-induced non-obstructive azoospermia rat mode was used to evaluate spermatogenic recovery ability of treated ADMSCs. Besides, the relative gene expression level was detected by reverse transcription PCR, quantitative RT-PCR. The relative protein expression level was detected by western blot (WB) and immunostaining analysis.

**Results:**

The results showed that ADMSCs co-cultured with TM4 cells under RA and T induction enhanced the formation of bigger and tightly packed MGLCs feature colonies in vitro. Moreover, the expression of male germ cell-related markers (Oct4, Stella, Ddx4, Dazl, PGP9.5, Stra8, and ITGα6) is significantly upregulated in TM4 cell-co-cultured ADMSCs in vitro and in busulfan-treated rat testis after injecting TM4 cell-treated ADMSCs for 2 months. Comparatively, the ADMSCs treated by TM4 cell with RA and T exhibited the highest expression of male germ cell-related markers. RA- and T-treated TM4 cell showed fewer dead cells and higher cytokine secretion than untreated groups. The protein expression level of TGFβ-SMAD2/3, JAK2-STAT3, and AKT pathways in ADMSCs co-cultured with TM4 cells under RA and T was higher than others. Whereas, downregulation of male germ cell-related marker expression subsequently inhibited the phosphorylation of SMAD2/3, JAK2, STAT3, and AKT.

**Conclusion:**

These results suggested that TM4 cells could efficiently stimulate in vitro generation of MGLCs during co-culturing of ADMSCs under RA and T treatment. Conclusively, the ADMSCs co-cultured with TM4 cell under RA and T induction stimulate the efficient generation of MGLCs in vitro through activating TGFβ-SMAD2/3, JAK2-STAT3, and AKT pathways. Among them, JAK2-STAT3 and AKT pathways are being first reported to show involvement of in vitro generation of MGLCs during ADMSC co-culturing with SCs.

**Electronic supplementary material:**

The online version of this article (10.1186/s13287-019-1181-5) contains supplementary material, which is available to authorized users.

## Introduction

Spermatogenesis is a complex and regular process of cell differentiation. Multiple genes and hormones regulate the whole process that starts from the division of spermatogonia cells, meiosis of spermatocytes to the metamorphosis and maturation of spermatocytes. Even minor damage in this process can cause sperm abnormalities and leads to infertility, which is a major medical problem that affects 10–15% couples worldwide [1]. Understanding the mechanism of spermatogenesis is a prerequisite for elucidating the molecular mechanisms of male infertility.

In the recent years, the in vitro generation of male germ cells from stem cells may provide an experimental platform for understanding germ cells development, molecular and cellular mechanisms, and treatment of male infertility [2, 3]. However, the prerequisite is to establish a suitable culture system to generate male germ cells from stem cells [4]. Consequently, a lot of research work has been performed and worthy progress has been made in this filed [4]. The most investigated stem cells are embryonic stem cells (ESCs), induced pluripotent stem cells (iPSCs), and spermatogonial stem cells (SSCs) [4, 5]. Mature spermatozoa have been reported to generate from these stem cells; nevertheless, human’s functionally intact mature spermatozoa cannot be easily obtained [4]. In addition, using these cells have some limitation, such as ESCs have ethical problems and their sources are limited, iPSCs have both oncological and genetic instability, and SSCs have low content in the testis, isolation, identification, and culturing difficulties in vitro [5, 6]. Mesenchymal stem cells (MSCs) do not have such problems in applications. These cells are easily obtained from mesenchymal tissue (such as bone marrow and adipose tissue) and are prone to propagate in vitro [7]. Furthermore, MSCs comprise of heterogeneous population of cells and contain pluripotent stem cells, namely multilineage-differentiating stress-enduring (Muse), which is the same as ESCs, which has the ability to differentiate into all cells from three germ layers spontaneously [8, 9]. The other non-Muse cell can secrete paracrine factors and affect other cell’s activities, including survival, immune regulation, cell migration, angiogenesis, proliferation, and antioxidation [10, 11]. As such, MSCs have been considered as a new cell source to generate MGLCs in vitro [12]. Moreover, the previous studies demonstrated that the MGLCs in pre-meiosis can generate during treatment of MSCs under suitable circumstances [13, 14, 15, 16, 17]. However, the efficient generation of male germ-like cell (MGLCs) by using MSCs is a difficult task [12, 18].

The regeneration potential of stem cells depends upon their surrounding microenvironment, including supporting cells and a growth factor milieu [19]. Sertoli cells (SCs) are the only adult cell in the seminiferous tubules that are necessarily required for testis formation and spermatogenesis. SCs along with their secreted factors are important components of testis microenvironment, which advance the progression of germ cells to spermatozoa [20, 21, 22]. Using a well-defined SC-gonocytes cell co-culture system has confirmed that the germ cells development may depend on interactions with adjacent SCs [23]. Results have shown that the presence of SC allowed germ stem cells to enter into meiotic progression [24], and increases GC survival and proliferation [25]. These findings indicated that SCs might enhance the generation of mature male germ-like cells during co-culturing of stem cells with SCs.

Adipose-derived mesenchymal stem cells (ADMSCs) possess all the characteristics of MSCs that are mentioned above. Moreover, it is more abundant and easy to obtain from adipose tissue with minimal damage and less patient discomfort. Therefore, they are considered as a good choice for cell therapy and application research [26]. In order to generate a large number of male germ-like cells (MGLCs), here, we efficiently generate MGLCs in vitro by co-culturing with TM4 Sertoli cells (TM4 cell) under RA and T treatment and further explain the possible mechanism pathways.

## Materials and methods

### Cells and animals

TM4 mouse Sertoli cells were purchased from American Type Culture Collection (ATCC). Male Sprague-Dawley (SD) rats (2–3 weeks) were obtained from Shanghai Jiesijie Experimental Animal Co., Ltd. (Shanghai, China). Animal experiments were performed according to the guidelines issued by the Institutional Animal Care and Use Committee of the Chinese Academy of Agricultural Sciences (CAAC).

### Rat ADMSC isolation, culturing, and identification

ADMSCs were isolated from 3-week male Sprague-Dawley (SD) rats and were identified to have osteogenic and adipogenic differentiation potential [27]. Passage-3 (P3) ADMSCs were used in the experiments.

### Mitomycin C inactivated TM4 cell

TM4 mouse Sertoli cells (TM4) (ATCC, Rockefeller, MD, USA) were plated at cell density of 2500 cells/cm^2^. At near confluence, cells were rinsed three times with phosphate buffer saline (PBS) and then were incubated with 10 μg/mL mitomycin C at 37 °C for 1 h. After that, cells were rinsed four times with PBS to completely remove mitomycin C. Then, cells were harvested and total cells were counted by using Countstar automatic counting cell analyzer IC1000 (Ruiyu, Shanghai, China). Cell suspensions were used to co-culture with ADMSCs.

### Stimulation of TM4 cell on the generation of MGLCs in vitro

In order to confirm the enhancement and mode of using SCs on the generation of MGLCs during co-culturing of ADMSCs with SCs in vitro, three experimental groups were investigated: treatment with RA and T (group DM), combination of RA and T with indirect co-culturing with mitomycin C inactivated TM4 cell (group IC), and combination of RA and T with direct co-culturing with mitomycin C (group DC); ADMSCs were treated by RA and T as control (Additional file 1: Figure S1). When passage-3 (P3) ADMSCs reached the confluence of 80–90%, these cells were collected and used to generate into MGLCs by the above mentioned three methods. These were confirmed by assessing the changes in cell morphology, expressions of male germ cell (MGCs)-related markers by quantitative real-time PCR, western blotting, and immunocytochemistry. ADMSCs after treating by these three methods for 21 days were injected into busulfan-treated recipient rat testes (Additional file 1).

### Transplantation of ADMSCs into busulfan-treated recipient rat testes

Untreated ADMSCs (group GM) and in vitro treated ADMSCs by different methods (groups DM, IC, and DC) were labeled by PKH26 Red Fluorescent and then were transplanted into busulfan-treated recipient rat testes; only medium injected testis was used as negative control, and non-injected testis was used as real control. After 2 months of transplantation, the survival of transplanted cells in seminiferous tubules was observed by inverted fluorescence microscopy. The spermatogenesis recovery capability of ADMSCs from different groups was compared by evaluating expressions of MGC-related markers by quantitative real-time PCR and immunohistochemistry (IHC).

### Busulfan-induced xenograft model

Busulphan was used to deplete endogenous male germ cell in testis for stem cell transplantation [28]. Four-week-old male SD rats were divided into three treatment groups (20 mg/kg, 40 mg/kg, and (20 + 20) mg/kg). In order to optimize the method of busulfan-induced xenograft model, we treated the rats with either intraperitoneal injection of 20 mg/kg or 40 mg/kg busulfan once, or 20 mg/kg busulfan twice with 14 days of interval, and at the same time, rats were treated with DMSO as control. According to the results of histological morphology and size of testicle, as well as fatality rate of rat after being treated by busulfan for a month (Additional file 1: Figure S4), we selected the best group ((20 + 20) mg/kg) to establish a busulfan-induced xenograft model. Four-week-old male Sprague-Dawley (SD) rats (*n* = 30) were treated with busulfan at least 4 weeks before donor cell transplantation.

### PKH26 labeling

In vitro induction and non-induction of ADMSCs labeled with PKH26 Red Fluorescent (Sigma-Aldrich, MO, USA) was performed as per the instructions. Labeling was qualitatively verified by fluorescence microscopy using Invitrogen™ EVOS™ FL Auto Imaging System (Life Technology, MA, USA). More than 95% cells labeled with the dye can be identified.

### Cell transplantation

In order to investigate effect of TM4 cell treatment in vitro on spermatogenesis recovery capability of ADMSCs, six experimental groups were initiated. Four treatment groups: in vitro untreated—ADMSCs injected (GM), RA and T treated in vitro for 21 days—ADMSCs injected (DM), combination of RA and T indirectly co-cultured with TM4 cell in vitro for 21 days treatment—ADMSCs injected (IC), combination of RA and T directly co-cultured with TM4 cell in vitro for 21 days treatment—ADMSCs injected (DC). Two control groups: only medium injected (M) and non-injected (NC). Cells harvested from each group were then injected into four testicles from different receptor rats. Twelve busulfan-treated SD rats were divided into four groups with the same treatment for each group. There were three rats in each group, in which three testes from two rats were injected with treated cells from IC, DC, and DM groups, respectively, and the other testis were used as the non-injected control (NC). Two testes from other rats were injected with untreated ADSCs (GM) and serum-free DMEM/F12 (M), respectively. The seminiferous tubules of recipient rat were filled with approximately 100 μL/testis of donor cell suspension (containing 1 × 10^6^ labeled cells) by injection through the recipient testis using insulin needle (JET BIOFIL, Guangzhou, China) as described previously [29, 30]. After 8 weeks, recipient rat were killed and all testes were harvested, and examined by biopsy, immunohistochemistry (IHC) and quantitative real-time PCR (qRT-PCR).

### Survival of transplanted cells in seminiferous tubules analysis

Survival of transplanted cells in seminiferous tubules was analyzed after 2 months of cell transplantation. Testicles were dissected out and decapsulated, seminiferous tubules were dispersed gently in PBS containing petri dish, and the live tissue was visualized under confocal laser scanning microscopy (Nikon AIR, Japan).

### Synergistic promoting effect of TM4 cell, RA, and T

Using Transwell insert co-culture system, we confirmed that TM4 cell could enhance the subpopulations of ADMSCs to represent MGCs-related markers in vitro under RA and T induction through paracrine modes of action. To understand the synergistic promoting effect of TM4 cell, RA, and T on the generation of MGLCs during co-culturing of ADMSCs, five experimental groups were investigated: (1) treatment with RA and T (control), (2) treatment with TM4 cell-conditioned medium (group TCCM), (3) treatment with TCCM, RA, and T (group TCCM+RA+T), (4) indirect co-culturing with mitomycin C inactivated TM4 cell (group TM4 cell), and (5) combination of RA and T with indirect co-culturing with mitomycin C inactivated TM4 cell (group IC). After 21 days of treatment, the expressions of MGC-related markers were assessed by quantitative real-time PCR, western blotting, immunocytochemistry, and flow cytometry (FCM) analysis (Additional file 1).

### Preparation of TM4 cell-conditioned medium (TCCM)

Passage 5–12(P5–12) TM4 cells were inoculated into T75 T flasks with density of 2 × 10^4^cells/cm^2^ and cultured in DMEM/F12 medium containing 5% FBS. At near confluence, cells were raised three times with PBS and then were incubated with serum-free DMEM/F12 medium. The medium was collected after 3 days of incubation and was stored as conditioned medium after 10 min centrifugation for 2000*g* at 4 °C.

### Retinoic acid and testosterone stimulate cytokines secretion from TM4 cells

To study the simulation effect on cytokines secretion of TM4 cells, TM4 cells were treated with RA and T. TM4 cells without RA and T treatment were used as a control. Mitomycin C inactivated passage10 TM4 cells were plated at cell density of 3 × 10^4^ cells/cm^2^ in a six-well plate and treated with and without 10^−5^ M, RA, and 2 μM T for 3 days. Morphological changes were observed every day using a phase contrast microscope, real-time quantitative RT-PCR, and western blot which were used to detect the genes and protein expression level of TM4 cells grown under different culture conditions on day 3.

### Pathways analysis

ADMSCs were treated by (1) RA and T (control) and (2) combination of RA and T with indirect co-culturing with mitomycin C inactivated TM4 cell for 21 days. The quantitative protein expression of pathways such as Wnt/β-catenin, mitogen-activated protein kinases (MAPKs), ERK1/2, p38 and JNK, TGFβ/SMAD2/3, Janus kinase-signal transducer and activator 3 of transcription (JAK/STAT3), and PI3K/Akt in ADMSCs from the two groups after 3 days and 21 days were evaluated by western blot. TGFβ/SMAD2/3, JAK2/STAT3, and PI3K/AKT signaling pathways were found to be significantly affected. These signaling pathways were further analyzed by corresponding signal pathway inhibitors.

To validate signaling pathway, indirect TM4 cell co-cultured ADMSCs were treated with TGFβ/SMAD2/3 signaling pathway inhibitor SB431542 (Selleck, USA), PI3K/AKT signaling pathway inhibitor LY294002 (Selleck, USA), and JAK/STAT3 signaling pathways inhibitor ruxolitinib (Selleck, USA) and niclosamide (Selleck, USA) for 21 days, respectively. Briefly, 2 × 10^5^ cells ADMSCs and 4 × 10^5^ cells mitomycin C inactivated TM4 cells were co-cultured in a six-well Transwell chamber culturing in basal medium, and TM4 cells were in the upper side of the chamber. After 2 days of co-culturing, medium was replaced by differential medium containing either 0.25 and 0.5 μM SB431542, 2.5 and 5 μM LY294002, 5 and 12.5 μM Ruxolitinib, or 0.25 and 0.5 μM Niclosamide. Cells without inhibitor treatment were used as control, and medium were changed after every 3 days. On day 21, AMDSCs were collected and the mRNA expression of MGCs-related marker in the treatment group was compared with the control group by qRT-PCR, and the protein expression of MGC-related markers as well as key components of signaling pathway in the treatment group was compared with the control group by western blotting (WB) (Additional file 1).

### Total RNA extraction and quantitative real-time PCR

Genes were assessed by using SYBR Premix Ex Taq II reagent Kit (Tli RNaseH Plus) (Takara, Dalian, China). Briefly, total RNA extraction was performed by using total RNA isolation kit RP5611 (Bioteke, Beijing, China). RNA content and purity were detected by NanoDrop 2000 ultra-micro spectrophotometer (Thermo Fisher Scientific, Cambridge, MA, USA). After erasing genomic DNA, the first-strand cDNA was obtained by using reverse transcription kit PrimeScript RT reagent Kit RR047A (Takara, Dalian, China). Then, the CFX96 Touch™ Fluorescence quantitative real-time PCR system (Bio-Rad, Hercules, CA, USA) was used to detect the relative expression of genes. The mRNA relative expression was quantified by 2^−△△Ct^ method. The genes and primers used in the study are mentioned in Additional file 1: Table S1.

### Western blotting analysis

After the extraction of protein, western blotting was performed by using standard protocols, as previously described by our group [31]. Sources of primary antibodies for Western blotting are listed in Additional file 1: Table S2.

### Statistical analysis

Statistical analysis was performed using SPSS 19 software (IBM, Amon, NY, USA). All statistical values are presented as means ± standard deviation (SD). Univariate comparisons of means were evaluated by using the Student’s test (*t*), *P* < 0.05 was considered statistically significant. The data shown in the figures are representative experiments performed in triplicate.

## Results

### Sertoli cells act as a stimulation factor to enhance the generation of male germ-like cells during co-culturing of rat adipose-derived mesenchymal stem cells with SCs in vitro

**Fig. 1:**
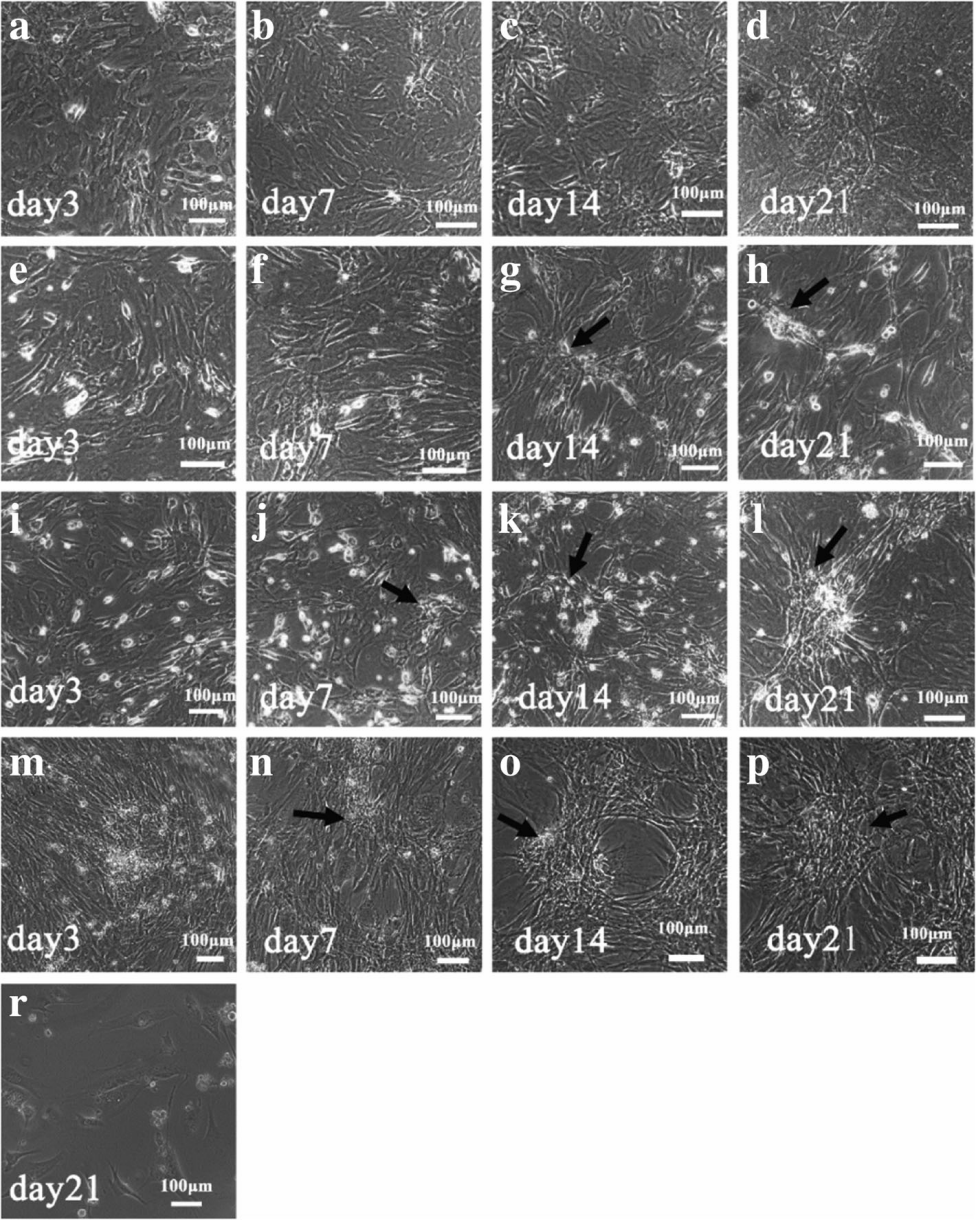
Morphology changes in adipose-derived mesenchymal stem cells (ADMSCs) in vitro treated by different methods on days 3, 7, 14, and 21. **a**–**d** ADMSCs treated with DMEM/F12 medium containing 10% FBS (GM). **e**–**h** ADMSCs treated with retinoic acid (RA) and testosterone (T) (DM). Cells stopped proliferation and formed loose cell clones on induced 14 days and 21 days (black arrow). **i**–**l** ADMSCs were indirectly co-cultured with TM4 cell in combination with RA and T treatment (IC). Cells stopped proliferation and formed loose cell clones on induced 7 days, tightly packed and flat clones were observed on induced 21 days (black arrow). **m**–**p** ADMSCs directly co-cultured with TM4 cell in combination with RA and T treatment (DC). Cells stopped proliferation and formed loose cell clones on induced 7 days; the clones are tightly packed up to 14 days (black arrow). **r** Morphology of mitomycin C inactivated TM4 cells on day 21

The efficient generation of MGLCs during co-culturing of rat ADMSCs (rADMSCs) with TM4 cells in vitro was investigated under RA and T induction (Additional file 1: Figure S1). The morphological changes in rADMSCs from different treatment groups were observed. Compared with non-treated ADMSCs, RA- and T-treated ADMSCs lost their spindle-like shape and generated colonies with MGLCs features during the process (Fig. 1). When compared to without TM4 cells co-cultured-ADMSCs, the colonies in TM4 cells co-cultured-ADMSCs appeared earlier; in addition, these colonies were tightly packed and became flat up to 21 days (Fig. 1e–p). However, the morphological changes showed no significant difference between cells in IC and DC groups (Fig. 1i–p). Morphologically, it indicates that TM4 cell can promote rADMSCs for giving rise to MGLCs.

**Fig. 2:**
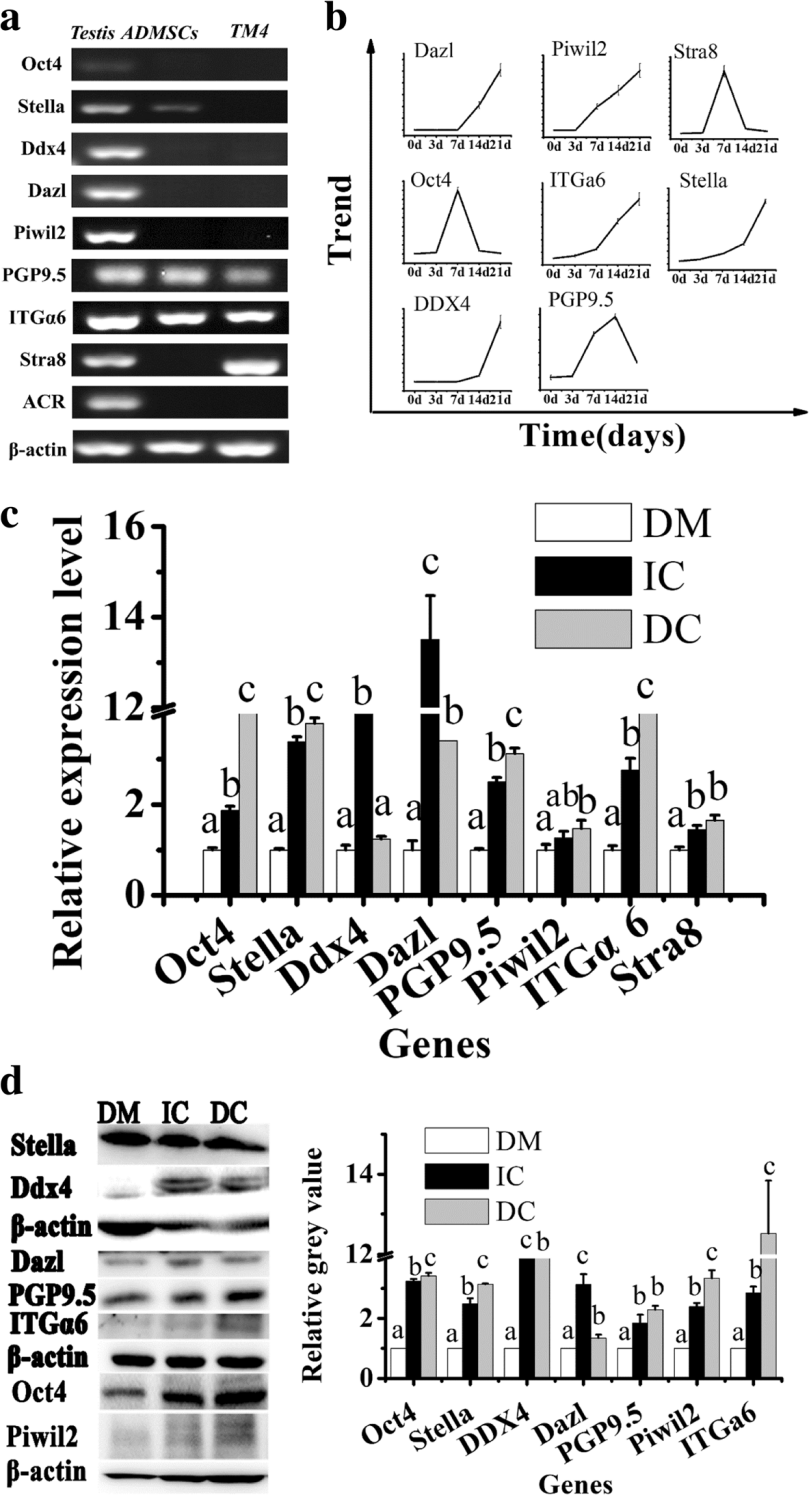
mRNA and protein expression of germ cell (GC)-related markers in the testis of male SD rats, TM4 cells, and ADMSCs from different culture groups. **a** Expression of genes on the testis of male SD rats, ADMSCs, and TM4 cells by RT-PCR. **b** Describing the trend of male germ cell-related markers expression in co-cultured ADMSCs giving rise to male germ-like cells (MGLCs) process. The expression of genes, including Stella, Ddx4, Dazl, Piwil2, and ITGα6, reached peaks at 21 days, while genes, such as Oct4, PGP9.5, and Stra8, reached peaks at 14 days and then downregulated. **c**, **d** Relative genes and protein expression in induced ADMSCs from three modes by qRT-PCR and western blotting on differentiated 21 days, **c** mRNA; **d** protein; individual induction (white bar), indirect co-culture with TM4 cells (black bar), and direct co-culture with TM4 cells (gray bar). Bar graphs represent mean ± SD (*n* = 3 per group). The results are presented as a mean ± SEM of three duplicate runs. Error bars in charts represent the corresponding standard deviations. *Y*-axis indicated the relative mRNA value normalized to induced ADMSCs from DM group mRNA level (white bar). Different letters on the bar graph indicate significant differences between the groups at the 0.05 level (*n* = 3)

In order to confirm the generation of MGLCs during co-culturing of rADMSCs, gene expressions of GC-related markers such as primordial germ cell (PGC)-related markers (Oct4, Stella, DDX4), spermatogonia and SSC-related markers (Dazl, PGP9.5, Piwil2, ITGα6, Stra8), and meiosis and post-meiosis male GC-related markers (ACR) in rADMSCs were detected by RT-PCR and quantitative RT-PCR (qRT-PCR). These results showed that Oct4, Stella, PGP9.5, and ITGα6 gene expression level in untreated rADMSCs was lower, and the expression of DDX4, Dazl, Piwil2, Stra8, and ACR genes could be used to distinguish ADMSCs from MGLCs (Fig. 2a, Additional file 1: Table S3). All the genes of GC-related markers (except ACR) can be detected in without TM4 cells co-cultured ADMSCs on day 14 (Additional file 1: Figure S2A), while it can be detected in with TM4 cell co-cultured ADMSCs on day 7 (Additional file 1: Figure S2B and C, Table 1). After treating with RA and T, the gene expression of GC-related markers in ADMSCs was all upregulated and the tendencies were roughly the same (Additional file 1: Figure S2). Except Oct4, PGP9.5, and Stra8, all the gene expression reached a peak level on day 21 (Fig. 2b). Then, we compared the gene and protein expression of male GC-related markers in ADMSCs after being treated by different methods on day 21 by qRT-PCR, WB, and immunostaining analysis. Results have shown that the gene expressions in ADMSCs from TM4 cell co-cultured groups are significantly higher than those from without TM4 cells co-cultured group (Fig. 2c). Compared with direct TM4 cell co-culture group, DDX4 and Dazl gene expression were significantly upregulated in the indirect co-culture group, while Stella and ITGα6 gene expression were significantly downregulated in the IC group (Fig. 2c). Results from western blotting and immunostaining analysis confirmed these specific biomarkers’ gene expression phenomenon (Fig. 2d and Additional file 1: Figure S3). Conclusively, the indirect TM4 cell co-culture group exhibited the highest efficiency for in vitro production of MGLCs in pre-meiosis during rADMSCs co-culturing with SCs.

**Table 1:** Expression pattern of Oct4, Stella, Ddx4, Dazl, PGP9.5, Piwil2, ITGα6, Stra8, and ACR

		Oct4	Stella	Ddx4	Dazl	PGP9.5	Piwil2	ITGα6	Stra8	ACR
ADSCs		+	+	–	–	+	–	+	–	–
Testis		+	+	+	+	+	+	+	+	+
DM	3 days	+	+	–	–	+	–	+	–	–
	7 days	+	+	–	–	+	+	+	+	–
	14 days	+	+	+	+	+	+	+	+	–
	21 days	+	+	+	+	+	+	+	+	–
IC	3 days	+	+	–	–	+	–	+	–	–
	7 days	+	+	+	+	+	+	+	+	–
	21 days	+	+	+	+	+	+	+	+	–
DC	3 days	+	+	–	–	+	–	+	–	–
	7 days	+	+	+	+	+	+	+	+	–
	21 days	+	+	+	+	+	+	+	+	–

### In vitro treatment of TM4 cell enhance spermatogenesis recovery capability of transplanted ADMSCs in busulfan-treated recipient rat testes

**Fig. 3:**
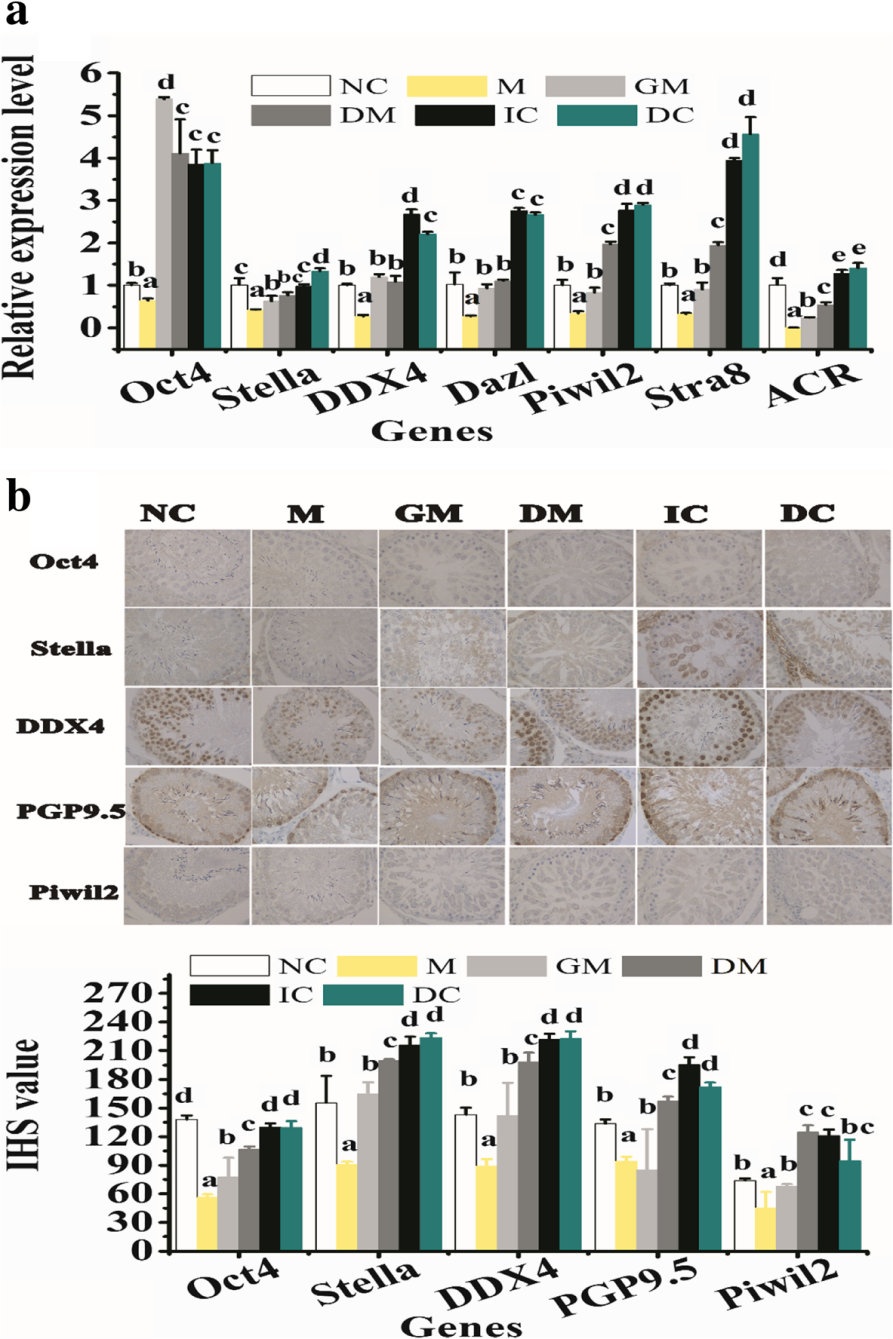
Relative male germ cell-related markers expression in recipient testis after different treatments for 2 months by qRT-PCR and immunohistochemistry (IHC) staining analysis. **a** Relative mRNA expression in recipient testis by qRT-PCR. *Y*-axis indicates the relative mRNA value normalized to testis from NC group (white bar) mRNA level. **b** Relative protein expression in sections of the testis by immunohistochemistry staining analysis. Bar graphs represent mean ± SD (*n* = 3 per group). Different letters on the bar graph indicate significant differences between the groups at the 0.05 level (*n* = 3)

PKH26-labeled rADMSCs from GM, DM, IC, and DC groups on day 21 were transplanted into the testis of an azoospermia rat model recipient to evaluate their potential spermatogenesis recovery. Transplanted rADMSCs can survive in busulfan-treated recipient rat testes for at least 2 months (Additional file 1: Figure S5). The expression of male GC-related markers in recipient rat testis from different treatment groups was evaluated by qRT-PCR and immunohistochemistry staining analysis. Reports have shown that in the testis of non-obstructive azoospermia mammals, the expression of genes related to male germ cells in the testis is upregulated along with the spermatogenesis recovery in busulfan-treated testis [32]. As compared to other treatment groups, the result showed that the gene expression of male GC-related markers in testicular injection of in vitro TM4 cell co-cultured rADMSCs was the highest, while there are no significant differences between TM4 cell indirectly co-cultured and directly co-cultured groups (Fig. 3a). Relative protein expression in sections of the testis by immunohistochemistry staining analysis results was further confirmed from this specific biomarkers’ gene expression phenomenon (Fig. 3b). These results indicated that co-culturing with TM4 cell before injecting into busulfan-treated recipient rat testes could enhance the spermatogenesis recovery capability of transplanted rADMSCs.

In this study, we have not investigated the fate of transplanted ADMSCs in the testis and the mechanism of spermatogenesis recovery. However, we found that the endogenous stem cell (namely very small embryonic-like stem cells (VSELs))-related markers such as Oct4, Oct4-A, Stella, SOX2, and Nanog can be detected in busulfan-treated recipient rat testes (Additional file 1: Figure S6) and Oct4, Stella, and ACR mRNA expression is significantly upregulated in the testes of busulfan-treated rat for 3 months rather than 2 months (Additional file 1: Figure S7). In addition, male germ cells were found in some seminiferous tubules of busulfan-treated rat testis using our methods (Additional file 1: Figure S4a). These results suggested that the native germ cells or stem cells might take part in self-repair spermatogenesis in chemoabalted testis.

### Synergistic effect of Sertoli cells, retinoic acid, and testosterone on in vitro generation of MGLCs during co-culturing of rat ADMSCs with SCs

**Fig. 4:**
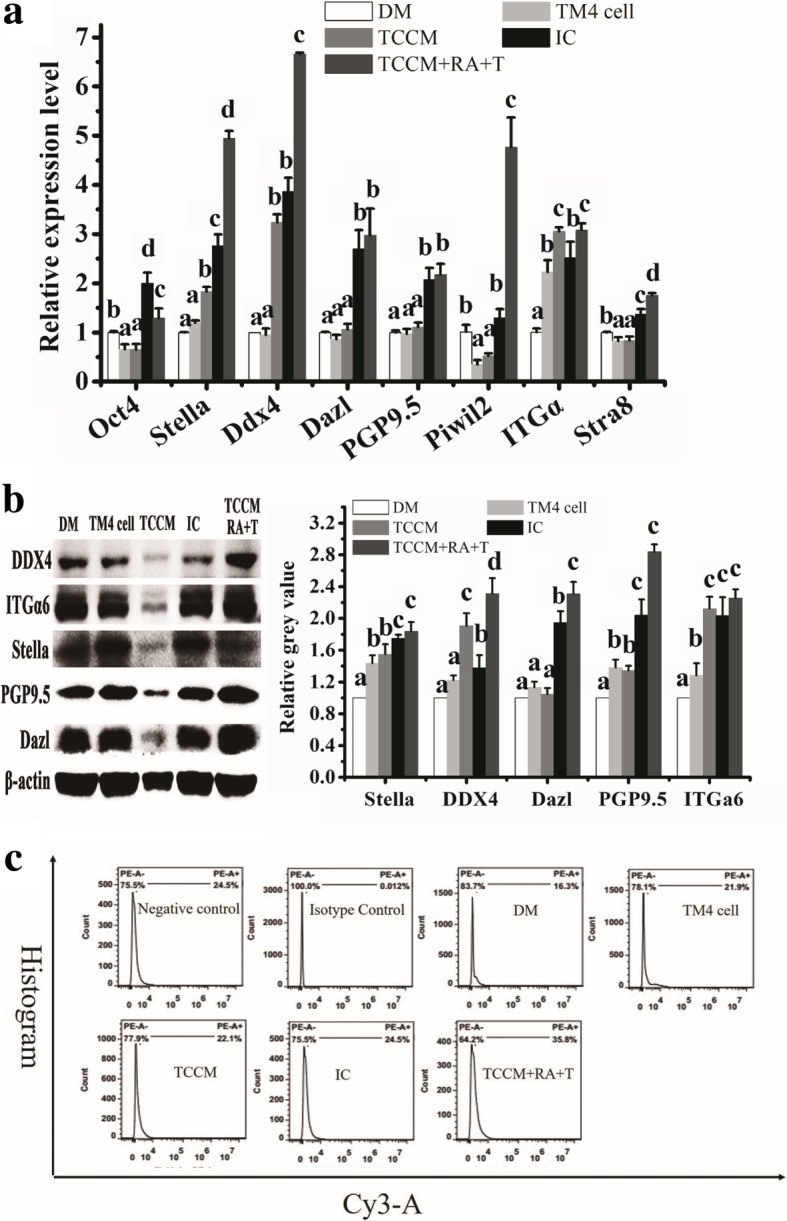
Male germ cell-related markers expression in ADMSCs after treating with RA, T, TM4 cell, and TM4 cell-conditioned medium (TCCM) for 21 days by qRT-PCR, WB and flow cytometry (FCM) analysis. DM: treatment with RA and T (white bar); IC: combination of RA and T treatment and indirect co-culture with TM4 cell (black bar); TM4 cell: indirect co-culture with mitomycin C inactivated TM4 cell (low gray bar); TCCM: treatment with TCCM (gray bar); TCCM+RA+T: treatment with TCCM, RA and T (dark gray bar). **a** Relative mRNA expression in ADMSCs by qRT-PCR analysis. *Y*-axis indicates the relative mRNA value normalized to ADMSCs from DM group (white bar). **b** Relative protein expression in ADMSCs by WB analysis. *Y*-axis indicates the relative gray value normalized to ADMSCs from DM group (white bar). **c** DDX4 expression in ADMSCs by FCM analysis. Bar graphs represent mean ± SD (*n* = 3 per group). Different letters on the bar graph indicate significant differences between the groups at the 0.05 level (*n* = 3)

ADMSCs after being treated by mitomycin C inactivated TM4 cell, TCCM, RA, and T generated colonies with MGLCs features (Additional file 1: Figure S8) and expressed protein of GC-related markers, such as Stella, PGP9.5, ITGα6, DDX4, and Dazl (Additional file 1: Figure S9). Compared with mitomycin C inactivated TM4 cell, RA, and T alone treated group, the gene expression of GC-related markers in ADMSCs from combination of RA and T with indirect co-culturing with mitomycin C inactivated TM4 cell group is the highest. The same results were observed in ADMSCs treated with TCCM, RA, and T. Furthermore, the gene expression of GC-related markers in ADMSC treatment with TCCM is significantly higher than that of indirect co-culture with TM4 cell (Fig. 4a). Results from WB and FCM analysis confirmed these specific biomarkers’ gene expression phenomenon (Fig. 4b, c). As such, TM4 cell, RA and T, and TCCM have synergistic effect and could stimulate the potential of ADMSCs in vitro giving rise to MGLCs.

### Retinoic acid and testosterone improve TM4 cells’ viability and stimulate cytokines secretion from TM4 cells

**Fig. 5:**
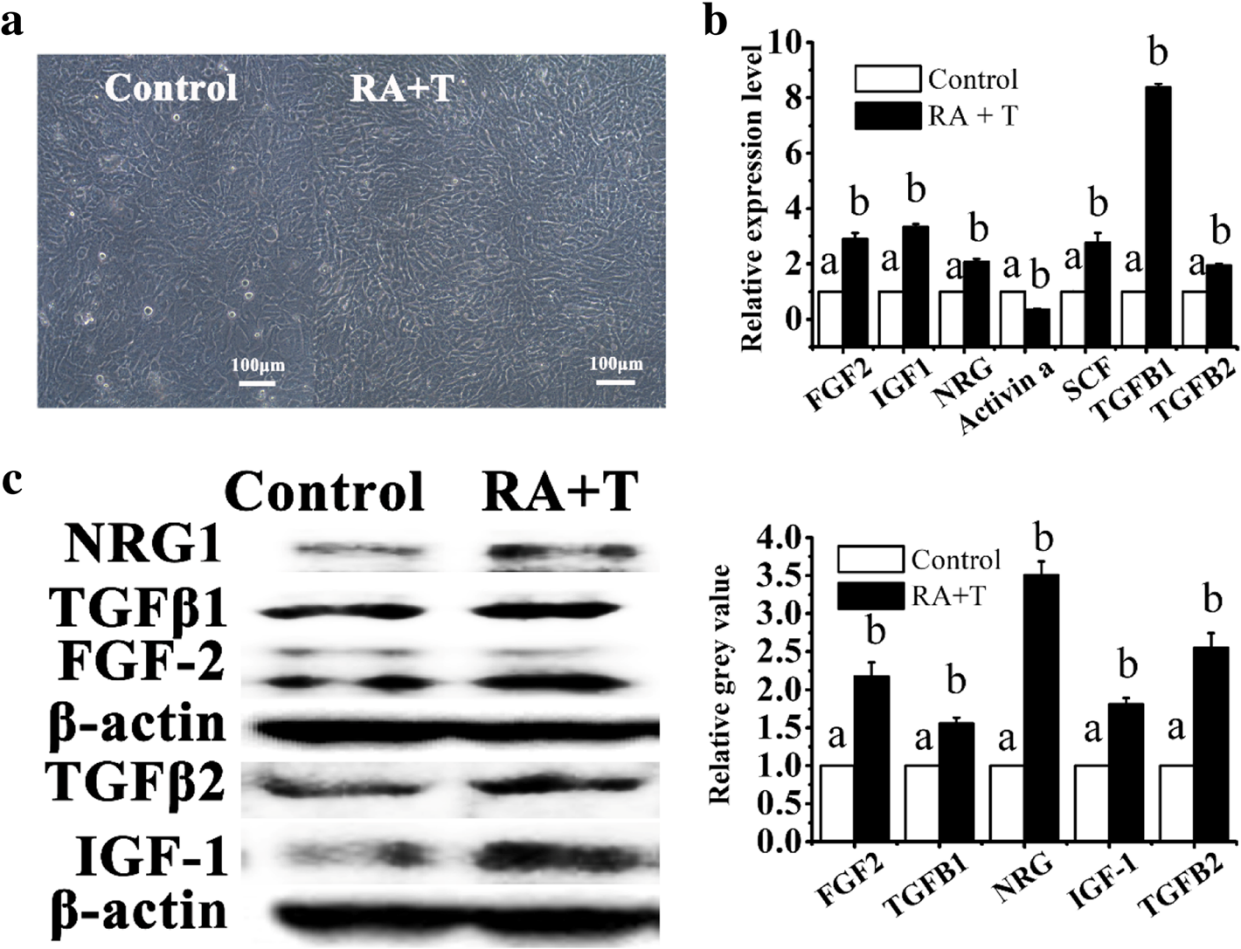
Examination of TM4 cells after treating with or without retinoic acid and testosterone on day 3. TM4 cells without retinoic acid and testosterone treatment were used as a control (white bar). **a** Morphologically, TM4 cells treated with RA and T showed fewer dead cells than the untreated group, (scale bar 100 μm). **b**, **c** The mRNA and protein expression of FGF2, NRG1, IGFI, TGFβ1, and TGFβ2 in RA- and T-treated TM4 cell is also upregulated than the untreated group. **b** Relative mRNA expression of cytokines genes by qRT-PCR. **c** Relative level of protein expression by western blot, quantitative analysis result by image-pro plus software. *Y*-axis indicates the relative mRNA value normalized to control group TM4 mRNA level. Bar graphs represent mean ± SD (*n* = 3 per group). Different letters on the bar graph indicate significant differences between the groups at the 0.05 level (*n* = 3)

Factors such as SCF, bFGF, IGFI, NRG1, Actα, TGFβ1, TGFβ2, and TGFβ3 have been reported to promote self-renewal and differentiation of SSCs [33, 34]. All these genes can be detected in TM4 cells by RT-PCR analysis (Additional file 1: Figure S10). TM4 cells inactivated by mitomycin C gradually died during the ADMSC co-culturing process, resulting in the decrease of paracrine factors (data not shown), and this might explain the lower ability to stimulate ADMSCs giving rise to MGLCs in indirect co-culture with mitomycin C inactivated TM4 cell group than treatment with TCCM group. To study the simulation effect on cytokine secretion of TM4 cells, TM4 cells were treated with RA and T. TM4 cells without RA and T treatment were used as a control. Morphologically, TM4 cells treated with RA and T showed fewer dead cells than the untreated group (Fig. 5a). The mRNA and protein expression of FGF2, NRG1, IGFI, TGFβ1, and TGFβ2 in RA- and T-treated TM4 cell is also upregulated than that in the untreated group (Fig. 5b, c). Therefore, the results manifest that the RA and T can improve TM4 cells’ viability and promote TM4 cells to secrete more trophic factors like cytokines including FGF2, NRG1, IGFI, TGFβ1, and TGFβ2. From another point of view, it is illustrated that the paracrine factor secreted by TM4 cell can stimulate the efficient generation MGLCs during co-culturing of ADMSCs with SCs in vitro.

### Sertoli cells enhance efficient generation of MGLCs during co-culturing of ADMSCs in vitro mainly via activation of TGFβ-SMAD2/3, AKT, and JAK2-STAT3 signaling pathways by phosphorylation

**Fig. 6:**
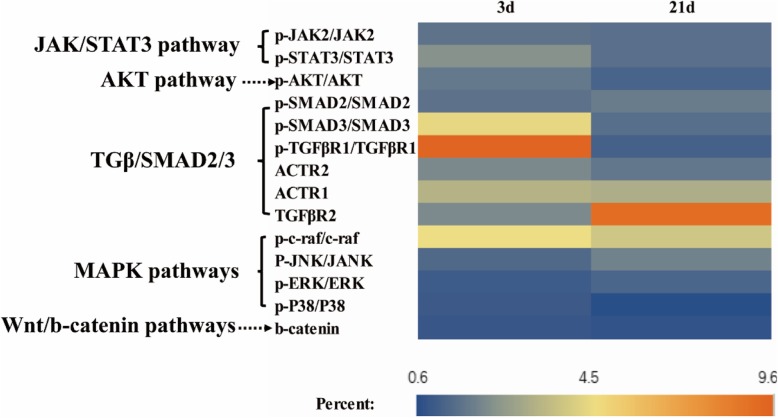
Heatmap of relative protein expression of key factors of TGFβ/Smad2/3, JAK2/STAT3, AKT, mitogen-activated protein kinases (MAPKs), ERK1/2, p38, JNK, and Wnt/β-catenin signaling pathway by western blot on differentiated 3d and 21d under DM and IC groups culture condition cells. Fold change of protein level of TGFβ-SMAD2/3, JAK2-STAT3, and AKT signaling pathways were larger (fold change more than 2) than that of ras/c-raf/JNK1/2, p38, and Wnt/β-catenin signaling pathways (fold change less than 2). The relative gray value normalized to DM group gray value

**Fig. 7:**
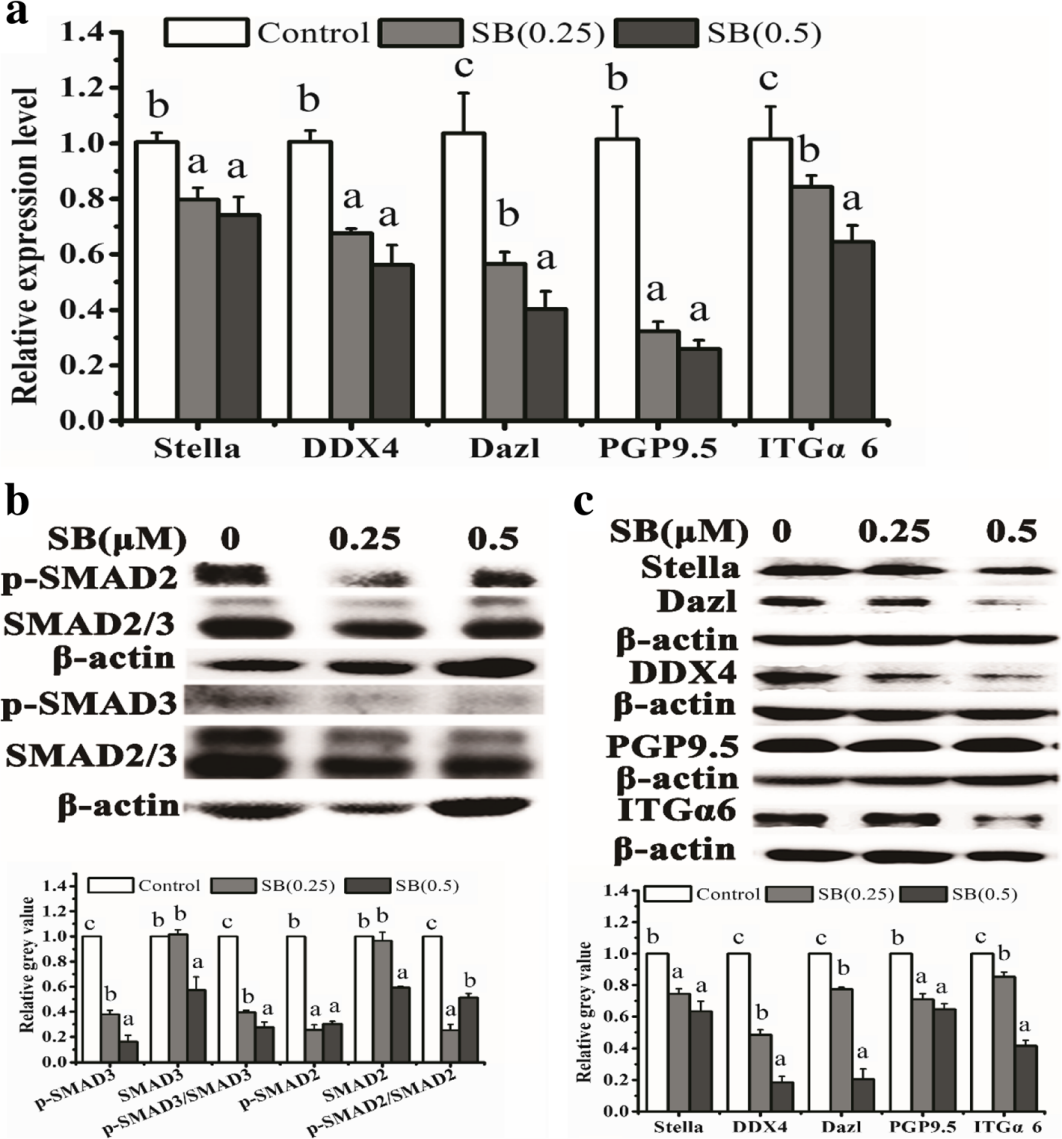
Expression of male GC-related markers in 0.25 and 0.5 μM SB431542-treated ADMSCs and untreated cells on 21d. **a** The mRNA expression of GC-related markers Stella, DDX4, Dazl, PGP9.5, and ITGα6 in ADMSCs were evaluated by qRT-PCR. **b** The protein expression of phosphorylation of SMAD2 (p-SMAD2), SMAD3, and total SMAD2/3 in ADMSCs was examined by western blot. **c** The protein expression of GC-related markers in ADMSCs were examined by western blot. Different letters on the bar graph indicate significant differences between the groups at 0.05 level (*n* = 3)

**Fig. 8:**
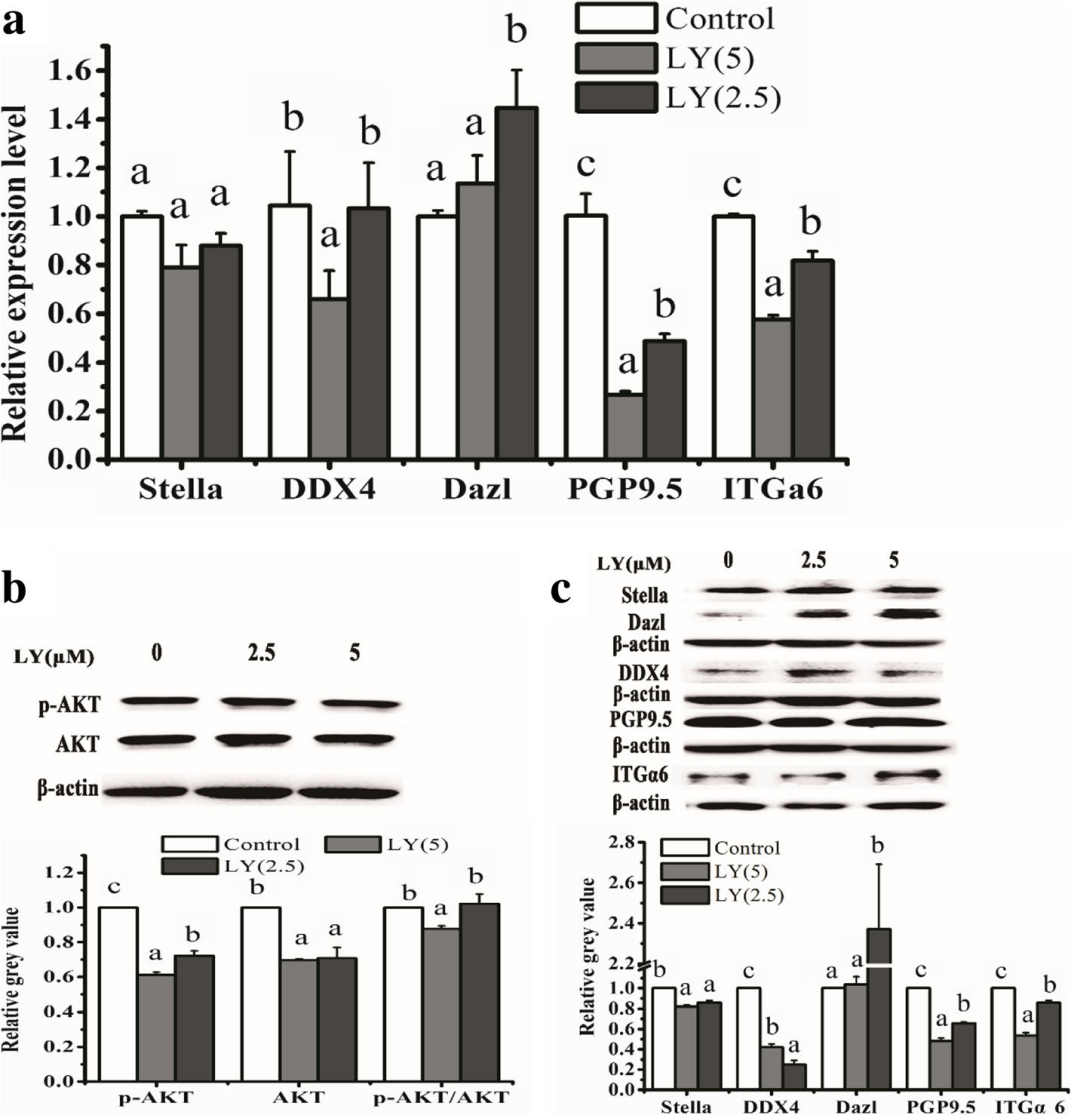
Expression of male GC-related markers in 2.5 and 5 μM SB431542-treated ADMSCs and untreated cells on 21d. **a** The mRNA expression of GC-related markers Stella, DDX4, Dazl, PGP9.5, and ITGα6 in ADMSCs were evaluated by qRT-PCR. **b** The protein expression of phosphorylation of AKT (p-AKT) and total AKT in ADMSCs was significantly downregulated after treating with 5 μM LY294002 for 21 days than that of untreated cells. **c** The protein expression of GC-related markers in ADMSCs were examined by western blot. Different letters on the bar graph indicate significant differences between the groups at the 0.05 level (*n* = 3)

**Fig. 9:**
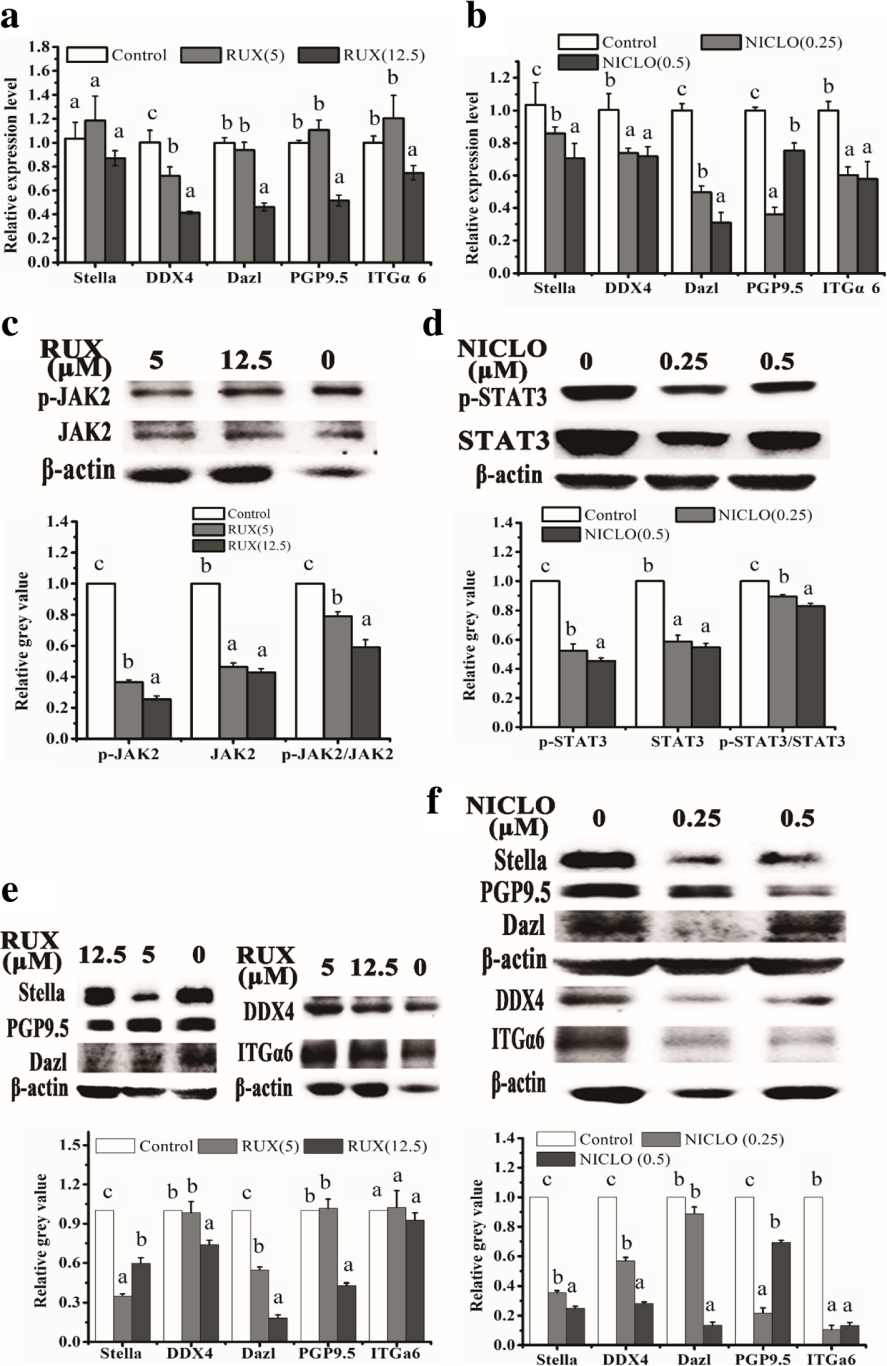
Expression of male GC-related markers in 5 and 12.5 μM ruxolitinib-treated ADMSCs and 0.25 and 0.5 μM niclosamide-treated ADMSCs and untreated cells on 21d. **a**, **b** The mRNA expression of GC-related markers Stella, DDX4, Dazl, PGP9.5, and ITGα6 in ADMSCs were evaluated by qRT-PCR. ADMSCs were treated with ruxolitinib (RUX) (**a**, **c**, **e**). ADMSCs were treated with niclosamide (NICLO) (**b**, **d**, **f**). **a**, **b** The mRNA expression of GC-related markers Stella, DDX4, Dazl, PGP9.5, and ITGα6 in ADMSCs were evaluated by qRT-PCR. **c** The protein expression of phosphorylation of JAK2 (p-JAK2) and total JAK2 in ADMSCs was examined by western blot. **d** The protein expression of phosphorylation of STAT3 (p-STAT3) and total STAT3 in ADMSCs was examined by western blot. **e**, **f** The protein expressions of GC-related markers in ADMSCs were examined by western blot. Different letters on the bar graph indicate significant differences between the groups at 0.05 level (*n* = 3)

TM4 cells secreted cytokines NRG1, FGF2, IGFI, Actα, TGFβ1, TGFβ2, and TGFβ3 which have been reported to activate the downstream factors including Wnt/β-catenin, mitogen-activated protein kinases (MAPKs), ERK1/2, p38 and JNK, TGFβ/SMAD2/3, Janus kinase-signal transducer and activator 3 of transcription (JAK/STAT3), and PI3K-Akt in other cells. We investigated the potential involvement of these signaling pathways in MGLCs generation during ADMSC co-culturing with SCs. In comparison with other groups (without TM4 co-cultured (DM group)), WB results show that the fold change of protein level of TGFβ-SMAD2/3, JAK2-STAT3, and AKT signaling pathways were larger (fold change more than 2) than that of ras/c-raf/JNK1/2, p38, and Wnt/β-catenin signaling pathways (fold change less than 2) in ADMSCs from TM4 co-cultured IC group (Additional file 1: Figure S11, Fig. 6). Therefore, we choose TGFβ-SMAD2/3, JAK2-STAT3, and AKT signaling pathways for further analysis by adding corresponding signal pathway inhibitors for 21 days, and the cells treated with DMSO were used as a control. The results have shown that in comparing DMSO treatment control group, SMAD2, SMAD3, AKT, JAK2, and STAT3 phosphorylation was remarkably inhibited in the corresponding signal pathway inhibitor group. Moreover, TM4 cells stimulated the generation of MGLCs during co-culturing of rADMSCs with SCs which were suppressed by pretreating cells with these inhibitors (Figs. 7, 8, and 9). In summary, these findings demonstrate that the TM4 cells stimulated rADMSCs giving rise to the generation of male MGLCs which was modulated by the activation of TGFβ-SMAD2/3, JAK2-STAT3, and AKT pathways.

## Discussion

In this study, we aim to develop a new system that is composed of TM4 cells, RA, and T for efficient generation of MGLCs during co-culturing of ADMSCs with SCs and to further unveil the possible mechanism involved in ADMSCs giving rise to MGLC process. Results indicated that TM4 cells could accelerate and enhance the generation of MGLCs in pre-meiosis during co-culturing of ADMSCs with SCs in vitro under RA and T treatment through activating TGFβ-SMAD2/3, JAK2-STAT3, and AKT signaling pathway. Our findings provide a new system to investigate the mechanism of male GCs development and an insight into the signaling networks regulating ADMSCs that gives rise to MGLCs process.

It is generally known that SCs play an important role in regulating spermatogenesis [35]. SCs along with their secreted factors regulate the progression of male germ cells maturation [35]. SCs and SC-condition medium (SCCM) are considered as an effective inducer to generate MGLCs during co-culturing of stem cells with SCs in vitro [16, 24, 36, 37, 38]. Similar with other stem cells [26, 34, 39], we find that co-culturing with SCs could stimulate the MGLCs generation during ADMSCs co-culturing with SCs (Figs. 1 and 2). However, the post-meiotic genes ACR were not detected in ADMSCs after co-culturing with TM4 cell combined with RA and T treatment for 21 days in this study, indicating the failure of ADMSCs to undergo meiosis. Therefore, more studies are required to explain the causes of failure to generate post-meiotic MGLCs during co-culturing of ADMSCs with SCs.

In addition, the present study provides further evidence that the treatment of MSCs before transplantation may affect the improvement of male infertility treatment effect [40, 41, 42]. We firstly demonstrated that TM4 cell co-cultured with ADMSCs directly or indirectly for 21 days in vitro could significantly enhance their spermatogenesis recovery ability in the testis of recipient rats after chemotherapy (Fig. 3). ADMSCs that represented higher MGLC-related markers in vitro exhibited strongest spermatogenesis recovery ability in busulfan-treated rat testis (Figs. 2 and 3). Our results are similar with earlier reports of Ghasemzadeh-Hasankolaei et al. In the previous study, the induction of ram BM-MSCs giving rise to MGLCs by RA and TGFβ1 was compared, and the results showed that TGFβ1 treatment for 21 days exhibited the highest efficiency for in vitro production of MGLCs as well as the highest capability for homing and colony formation in the testes [42]. However, no reports have been found about why the treatment of MSCs before injection could enhance the transplantation outcomes. In fact, MSCs are comprised of heterogeneous population of cells and contain a small amount of pluripotent stem cells which are known as Muse cells. Only these cells can spontaneously differentiate into cells from endodermal and ectodermal. Furthermore, these cells have strong sense of damage signals released by damaged/injured tissues and could migrate into injured tissues through bloodstream, differentiating in vivo into cells identical with the cells from the target cells after integration [9]. Moreover, previous study has shown that the local injected Muse cells could directly affect the tissue repair [43]. In addition, our results also suggest that the TM4 cell might stimulate the differentiation of Muse cells in ADMSCs into MGLCs and further might affect ADMSCs spermatogenesis recovery ability. However, future studies are needed to confirm this hypothesis.

At present, there is not very clear picture about the complete mechanism of highly efficient generation of MGLCs during co-culturing of rADMSCs with TM4 cells combined with RA and T treatment. Previously, some in vivo studies in mice have shown that the GCs originate from the proximal epiblast [44], while ADMSC cells originate from the mesoderm [45]. However, GCs specifically required paracrine signaling molecules, such as BMPs, which are derived from the extraembryonic ectoderm and visceral endoderm [46]. The multipotency of ADMSCs and their ability to generate MGLCs may be attributed to its inherent heterogeneity. Previous reports have shown that the subpopulation of very small embryonic-like (VSELs) was detected in bone marrow and other tissues [47]. VSELs express both pluripotent (Oct-4A, Sox2, and Nanog) and PGC-specific markers (Stella). As a result, it is easy to differentiate into three embryonic germ layers and spontaneously produce sperm and oocyte in vitro [47]. Studies on testicular VSELs in mouse models have shown that they have the ability to differentiate into sperm in vitro [48]. In the current study, we found that rADMSCs themselves express genes such as Oct-4A, Sox2, Nanog, and Stella (Fig. 1 and Additional file 1: Figure S6). These results suggested that the subpopulation of cells from rADMSCs such as pluripotent stem cells, namely multilineage-differentiating stress-enduring (Muse) cells, might take active part in the generation of MGLCs during co-culturing of rADMSCs with SCs.

In addition to the heterogeneity of ADMSCs, our result suggested that it maybe because of paracrine factors of TM4 cells possessing strong stimulating effects to generate MGLCs during co-culturing of ADMSCs with SCs under RA and T having the best cooperation effects through activating TGFβ-SMAD2/3, JAK2-STAT3, and AKT signaling pathway.

### Sertoli cells, RA, and T have a synergistic effect on stimulated male GLC generation during rADMSC co-culturing with SCs under RA and T induction

Retinoic acid (RA), an active metabolite of vitamin A, and T is a kind of androgen. Both are the key factors of the testis microenvironment. SCs have both retinoic acid receptor (RAR) and androgen receptors (ARs). Previous reports have shown that RA and T act on SCs and regulate their growth factor secretion to control and maintain in vivo spermatogenesis [20, 49, 50, 51]. Compared with the Sertoli cells without hormones, vitamins, and growth factors, the Sertoli cells in combination with hormones (e.g., T, FSH) or vitamins (e.g., RA) have been proven to upregulate post-meiotic genes and enhance the differentiation ability of stem cells [24]. Consistent with our finding that RA and T improved TM4 cells’ viability, promoted TM4 cells to secrete more trophic factors, and interacted with paracrine factors of TM4 cells that lead to synergistic stimulation of MGLC generation during ADMSC co-culturing with SCs in vitro (Figs. 4 and 5). Our results suggest that the RA and T improve TM4 cell viability and make them maintain high-factor secreting capacity, which results in high generation efficiency of MGLCs during ADMSC co-culturing with SCs under RA and T induction.

### TM4 cells along with RA and T co-stimulated rat ADMSCs to generate MGLCs in vitro through activating TGFβ-SMAD2/3, JAK2-STAT, and AKT pathways

It has been proven that the TGFβ/SMAD2/3, JAK2/STAT3, and PI3K/Akt signaling pathways are crucial for stem cell differentiation [34, 35, 52]and regulation of embryonic stem cell differentiation into male germ cells process [53, 54, 55, 56]. However, until now, limited information was found in their roles in regulation of ASCs (e.g., mesenchymal stem cells) into MGLCs differentiation process. Recently, Fang et al. reported that CD61 could induce cADMSCs to differentiate into primordial germ cell (PGC)-like cells by activating the TGFβ/SMAD2/3 signaling pathway [15]. However, our study suggested that the ADMSCs comprise of heterogeneous population of cells and contain pluripotent stem cells which might differentiate into germ cells. Moreover, our findings further demonstrated that except TGFβ-SMAD2/3 signaling pathway, activated JAK2-STAT3 and PI3K-AKT signaling pathways could also enhance the generation of MGLCs during rADMSCs co-culturing with SCs. We found that TM4 cells secrete multiple cytokines through paracrine way and may enhance the generation of MGLCs during rADMSCs co-culturing with SCs by activating TGFβ-SMAD2/3, JAK2-STAT3, and AKT signaling pathways (Figs. 6, 7, 8, and 9). The JAK-STAT signaling pathway is stimulated by cytokines and play essential role in development and cellular process, such as proliferation, differentiation, apoptosis, and immune regulation. Interleukin-10 (IL-10), bone morphogenetic protein 4 (BMP4), etc. could regulate osteogenic and dendritic cells differentiation of MSCs via JAK2-STAT3 signaling pathway [57, 58]. Furthermore, activated AKT regulates cell function by phosphorylating various enzymes, kinases, transcription factors, and other factors. The AKT signaling pathway play critical role for MSCs proliferation [27, 59], apoptosis [60], migration [61], and differentiation [62]. However, as far as we know, for the first time, we indicated that JAK2-STAT3 and AKT signaling pathway play a positive role for improving the generation of MGLCs during co-culturing of ADMSCs with SCs.

**Fig. 10:**
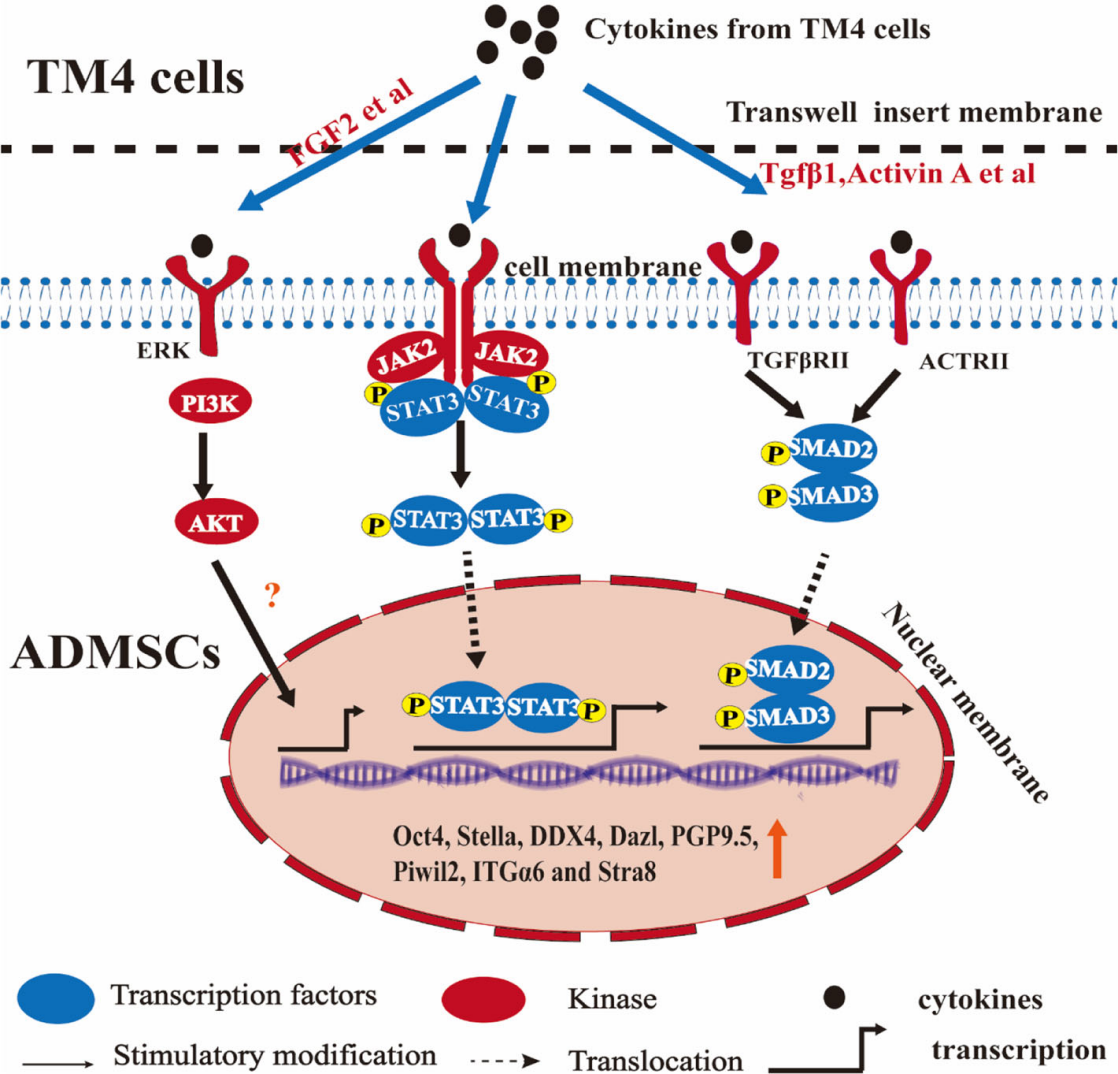
Schematic diagram of ADMSCs co-cultured with TM4 cells illustrates that the TM4 cells secrete multiple cytokines which upregulate the expression of germ cell (GC)-related markers in ADMSCs through activated TGFβ/Smad2/3, JAK2/STAT3, and AKT signaling pathways

Unfortunately, there is no further study which reveals the confirmed factors secreted by TM4 cells to stimulate the generation of MGLCs during co-culturing of rADMSCs with SCs through the above mentioned signaling pathways. Activin A and TGFβs regulated the differentiation of stem cells via TGFβ-SMAD2/3 signaling pathway [33], which have been reported to stimulate in vitro MGLCs differentiation [63, 64]. SCF, bFGF, IGFI, and NRG1 are able to promote self-renewal and differentiation of SSCs through PI3K-AKT signaling pathway [34]. BMP4 have shown to stimulate JAK2-STAT3 signaling pathway and affect the self-renewal of germline stem cells (GSCs) and MGLC differentiation [65, 66]. IGFI, which has demonstrated to activate JAK2-STAT3 signaling pathway [67], plays an important role in maintaining SSCs pluripotency and MGLCs differentiation [68]. Our finding shows that the TM4 cells might be responsible for expressing the genes of the above mentioned factors (Additional file 1: Figure S10). Therefore, we speculated that TM4 cells secreted cytokines such as FGF2, IGFI, NRG1, SCF, Actα, TGFβs, and BMP4 which might be responsible for generating MGLCs during co-culturing of rADMSCs with SCs through activating TGFβ-SMAD2/3, JAK2-STAT3, and AKT signaling pathways, as shown in Fig. 10. However, further researches are required to clearly illustrate which factors are responsible for stimulating the generation of MGLCs during co-culturing of ADMSCs with SCs, which can help us to analyze the mechanisms governing the generation of MGLCs during co-culturing of ADMSCs with SCs and to improve their generation efficiency.

## Conclusion

We have demonstrated the importance of microenvironment provided by SCs in co-cultured rADMSCs giving rise to MGLCs, which provides an efficient method for in vitro generation of MGLCs. Moreover, the SC-ADMSC co-culture system can be further used to study the hormones and growth factors that affect the generation of MGLCs during ADMSC co-culturing with SCs. In addition, the present study provides new methods to improve the spermatogenesis recovery ability of ADMSCs in azoospermic testis by co-culturing with SCs with RA and T treatment for 21 days in vitro. Finally, we confirm that paracrine factors from TM4 cells enhance MGLCs differentiation capacity through activation of TGFβ-SMAD2/3, JAK2-STAT3, and PI3K-AKT signaling pathways in a ADMSC co-culture mode, which provides a theoretical basis for further improving the efficiency of rADMSCs giving rise to MGLCs.

## Additional file

Additional file 1: Detailed materials and methods. (DOCX 8979 kb)
