# Supplementary material for: Efficient generation of male germ-like cells derived during co-culturing of adipose-derived mesenchymal stem cells with Sertoli cells under retinoic acid and testosterone induction
Source: Stem Cell Res Ther. 2019 Mar 13;10:91. doi: 10.1186/s13287-019-1181-5 (PMC6415496; doi:10.1186/s13287-019-1181-5)
Supplement: Supplementary file 1 — Detailed materials and methods. (DOCX 8979 kb) [file 13287_2019_1181_MOESM1_ESM.docx]

**Additional file**

**MATERIALS AND METHODS**

**Treatment with RA and T (Group DM):** P3 ADMSCs were collected and inoculated in 6-well plates with density of 2×10^5^ cells/well culturing in DMEM/F12 medium (Gibco, USA) containing 10% FBS (Sigma, USA) (basal medium). 2 days later, when ADMSCs were reached to 80% confluence, culture medium was replaced by DMEM/F12 medium containing 10% FBS, 10^-5^M retinoic acid (RA) (Sigma, USA) and 2 μM testosterone (T) (Sigma, USA) (differential medium), medium was changed every 3 days (Supplementary Figure 1A).

**Combination of RA and T treatment and indirectly co-culturing with TM4 cell (Group IC):** Rat ADMSCs were indirectly co-cultured with TM4 cell using 3 μm pore filters Transwell insert system (Corning Costar, Kennebunk, ME, USA) for 2 days. Briefly, P3 ADMSCs were collected and inoculated in 6-well plates with density of 2×10^5^ cells/well culturing in basal medium. At the same time, 4×10^5^ cells/well mitomycin C inactivated TM4 cells were seeded into 6-well plates on the upper side of the Transwell chamber and cultured in DMEM/F12 medium containing 5% FBS. When all cell adhere to the wall, upper side of the Transwell chamber were moved to the well. 2 days later, when ADMSCs were reached to 80% confluence, culture medium was replaced by differential medium, and medium was changed every 3 days (Supplementary Figure 1B).

**Combination of RA and T treatment with direct co-culturing with TM4 cell (Group DC):** Mitomycin C inactivated TM4 cells were seeded into 6-well plates with density of 4×10^5^ cells/well. When all cells adhere to the wall, P3 ADMSCs were collected and inoculated in the same 6-well plates with density of 2×10^5^ cells/well culturing in basal medium. 2 days later, culture medium was replaced by differential medium, and medium was changed every 3days (Supplementary Figure 1C).

**Treatment with TM4 cell-conditioned medium (group TCCM):** P3 ADMSCs were collected and inoculated in 6-well plates with density of 2×10^5^ cells/well culturing in DMEM/F12 medium containing 10% FBS. When ADMSCs were reached to 80% confluence, culture medium was replaced by 2×DMEM/F12 medium mixed with TCCM in a ratio of 1:1 supplemented with 10% FBS, and medium was changed every 3 days.

**Treatment with TCCM, RA and T (group TCCM+RA+T):** P3 ADMSCs were collected and inoculated in 6-well plates with density of 2×10^5^cells/well culturing in DMEM/F12 medium containing 10% FBS. When ADMSCs were reached to 80% confluence, culture medium was replaced by 2×DMEM/F12 medium mixed with TCCM in a ratio of 1:1 supplemented with 10% FBS, 10^-5^M RA, 2 μM T, and medium was changed after every 3 days.

**Indirect co-culturing with mitomycin C inactivated TM4 cell (group TM4 cell):** ADMSCs were co-cultured with mitomycin C inactivated TM4 cells by Transwell insert (the procedure is the same as that of IC group) under the growth medium.

**Validating signaling pathway:** TM4 cell indirect co-cultured ADMSCs were treated with TGFβ/SMAD2/3 signaling pathway inhibitor SB431542 (Selleck, USA), PI3K/AKT signaling pathway inhibitor LY294002 (Selleck, USA) and JAK/STAT3 signaling pathways inhibitor Ruxolitinib (Selleck, USA) and Niclosamide (Selleck, USA) for 21 days respectively. Briefly, 2×10^5^ cells ADMSCs and 4×10^5^ cells mitomycin C inactivated TM4 cells were co-cultured in 6-well Transwell chamber culturing in basal medium, and TM4 cells were in the upper side of the chamber. After 2 days of co-culturing, medium was replaced by differential medium containing either 0.25, 0.5 μM SB431542, or 2.5, 5 μM LY294002, or 5, 12.5μM Ruxolitinib, or 0.25, 0.5 μM Niclosamide. Cells without inhibitors treatment was used as control, and medium was changed after every 3 days. On day 21, AMDSC were collected and the mRNA expression of MGCs-specific marker in treatment group was compared with control group by qRT-PCR. Moreover, the protein expression of MGCs-specific marker as well as the key components of signaling pathway in treatment group were compared with control group by western blotting (WB).

**Table S1:** List of primers used in this study

| **Gene** | **Sequence** |
| --- | --- |
| **Oct4** | **F：**5‘ CCCAGCGCCGTGAAGTTGGA 3’  **R：**5‘ AGAACGCCCAGGGTGAGCCC 3’ |
| **Stella** | **F：**5‘ AAGATGAAGGGGACTCACCG 3’  **R：**5‘ GTCTCTGCTCAATCCGAACAAA 3’ |
| **Ddx4** | **F：**5‘ GGTGGTGGCCTTTTTGGTTC 3’  **R：**5‘ AAGCCTTTGTAGGCACCTCG 3’ |
| **Dazl** | **F：**5‘ CCCACAAAAGAAATCTGTGGACC 3’  **R：**5‘ GCACTGCCCGACTTCTTCTA 3’ |
| **Piwil2** | **F：**5‘ CCAGTTGGTGGAAAGGTAACGA 3’  **R：**5‘ AAAGGTCTAGGAGCTTGCGG 3’ |
| **PGP9.5** | **F：**5‘ CGGCCCAGCATGAAAACTTC 3’  **R：**5‘ TTATTGGCCACTGCGTGGAT 3’ |
| **ITGα6** | **F：**5‘ CACTCAGGTTCGAGTGACGG 3’  **R：**5‘ TCGTACCTAGAGCGCTTAAAGA 3’ |
| **Stra8** | **F：**5‘ AACAGCATCATCTCCGCACA 3’  **R：**5‘ AGAGGATCTCTTCCGGGGTG 3’ |
| **ACR** | **F：**5‘ GGCGTGTCACATCAACTAATG 3’  **R：**5‘ ATCCCCACGATCACAAAGG 3’ |
| **β-actin** | **F：**5‘ GGTGTGATGGTGGGTATG 3’  **R：**5‘ CAATGCCGTGTTCAATGG 3’ |
| **Oct4-A**[1] | **F：**5‘ GGCTGGACACCTGGCTTCAGA 3’  **R：**5‘ TGGTCCGATTCCAGGCCCA 3’ |
| **Sox2** | **F：**5‘ GAACTAGACTCCGGGCGATG 3’  **R：**5‘ CCCAGCAAGAACCCTTTCCT 3’ |
| **Nanog** | **F：**5‘ GAGCTATAAGCAGGTGAAGACC 3’  **R：**5‘ GGTTTCCAGACGCGTTCATC 3’ |
| **Gapdh** | **F：**5‘ GACATGCCGCCTGGAGAAAC 3’  **R：**5‘AGCCCAGGATGCCCTTTAGT 3’ |

Reference (Table S1)

1. Gonzalez RF, Allen L, Dobbs LG: **Rat alveolar type I cells proliferate, express OCT-4, and exhibit phenotypic plasticity in vitro.** *Am J Physiol Lung Cell Mol Physiol* 2009, **297:**L1045-1055.

**Table S2** List of antibodies used in western blotting.

| **Antibody** | **Dilution** | **Producer** | **Antibody** | **Dilution** | **Producer** |
| --- | --- | --- | --- | --- | --- |
| Oct4 | 1:1000 | Proteintech, USA | Phosphor SMAD2 | 1:1000 | CST, USA |
| DAZL | 1:1000 | Proteintech, USA | SMAD2 | 1:1000 | CST, USA |
| PGP9.5 | 1:1000 | Proteintech, USA | Phosphor SMAD3 | 1:1000 | SAB, China |
| ITGα6 | 1:1000 | Proteintech, USA | SMAD3 | 1:1000 | CST, USA |
| DDX4 | 0.5ug/mL | Biorbyt, UK | SMAD2/3 | 1:1000 | CST, USA |
| Stella | 1:200 | Santa, USA | Phosphor JAK2 | 1:1000 | SAB, China |
| Piwil2 | 1μg/mL | R&D, USA | JAK2 | 1:1000 | CST, USA |
| Phosphor c-raf | 1:1000 | CST, USA | Phosphor STAT3 | 1:1000 | SAB, China |
| c-raf | 1:1000 | CST, USA | STAT3 | 1:1000 | SAB, China |
| ERK1/2 | 1:1000 | CST, USA | Phosphor PI3K | 1:1000 | SAB, China |
| Phosphor ERK1/2 | 1:1000 | CST, USA | PI3K | 1:1000 | CST, USA |
| Phosphor p38 | 1:1000 | CST, USA | Phosphor AKT | 1:1000 | CST, USA |
| P38 | 1:1000 | CST, USA | AKT | 1:1000 | CST, USA |
| Phosphor JNK1/2 | 1:1000 | CST, USA | IGFI | 1:1000 | Proteintech, USA |
| JNK1/2 | 1:1000 | CST, USA | FGF2 | 1:1000 | Proteintech, USA |
| β-catenin | 1:1000 | CST, USA | TGFβ1 | 1:1000 | SAB, China |
| Phosphor TGFβR1 | 1:1000 | SAB, China | TGFβ1 | 1:1000 | SAB, China |
| TGFβR1 | 1:1000 | SAB, China | β-actin | 1:2500 | SAB, China |
| TGFβR2 | 1:2000 | SAB, China | Goat anti-Rabbit IgG Secondary antibody | 1:10000 | SAB, China |
| ACVR1 | 1:1000 | SAB, China | Rabbit anti-mouse IgG Secondary antibody | 1:1000 | R&D, USA |
| ACTR2 | 1:1000 | SAB, China |  |  |  |

**Table S3 The expression of germ cell (GC)-related markers among Testis, ADMSCs and TM4 cells by qRT-PCR, water as negative control, the data is C_t_ mean ± SD**. All genes were detected in Testis. Ddx4, Dazl, Piwil2, Stra8 and ACR genes cannot be detected in ADMSCs. Except PGP9.5, ITGα6 and Stra8, other genes cannot be detected in TM4 cells.

| **The male germ cells specific markers** | | | | |
| --- | --- | --- | --- | --- |
| **gene** | **Testis** | **ADSCs-male** | **TM4 cells** | **Water** |
| **Oct4** | 30.61±0.02 | 34.62±0.21 | 36.13±0.02 | 37.1±0.04 |
| **Stella** | 29.485±0.16 | 31.11±0.02 | 36.08±0.72 | 37.1±0.28 |
| **DDX4** | 26.79±0.04 | 35.43±0.06 | 34.16±0.81 | 35.55±0.04 |
| **Dazl** | 24.15±0.08 | 34.56±0.03 | 34.09±0.41 | 36.36±0.44 |
| **PGP9.5** | 20.37±0.269 | 23.89±0.31 | 29.96±0.26 | 38.27 ±0.021 |
| **Piwil2** | 26.49±0.28 | 33.82±0.30 | 34.65±0.47 | 34.18±0.35 |
| **ITGα6** | 21.87±0.23 | 24.77±0.04 | 26.09±0.15 | 35.9±0.41 |
| **Stra8** | 26.35±0.25 | 36.83±0.08 | 33.72±0.33 | 36.79±0.12 |
| **ACR** | 33.50±0.44 | 35.79±0.46 | 40±0.00 | 40±0.00 |
| **Gapdh** | 18.80±0.77 | 15.7±0.04 | 14.17±0.02 | 36.9±0.22 |

**Figure S1**

**
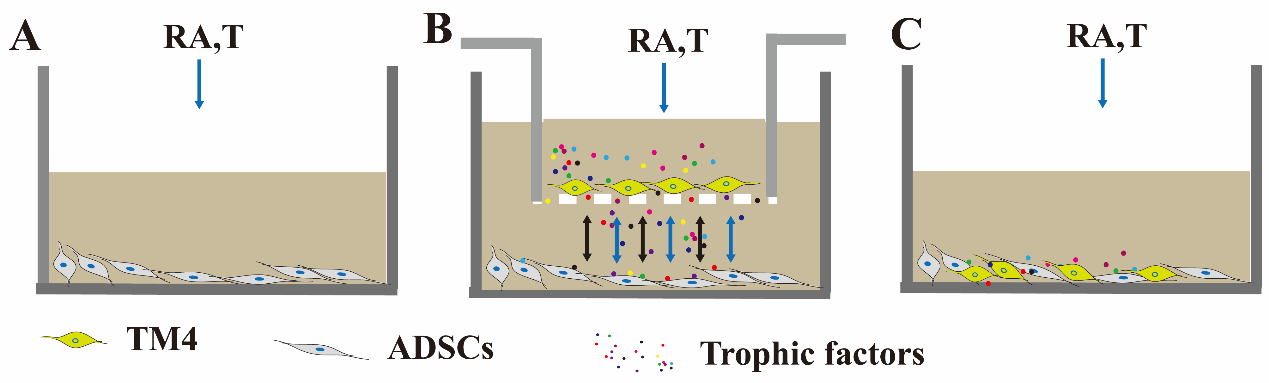
**

**Figure S1 Schematic diagram showing induction of adipose-derived mesenchymal stem cells (ADMSCs) giving rise to male germ-like cells (MGLCs) by three modes.** (A). Individual induction. (B). Interactions between the upper and lower compartments of indirect co-culture system. (C) Direct co-culture with TM4 cells. Retinoic acid (RA), testosterone (T).

**Figure S2**

**
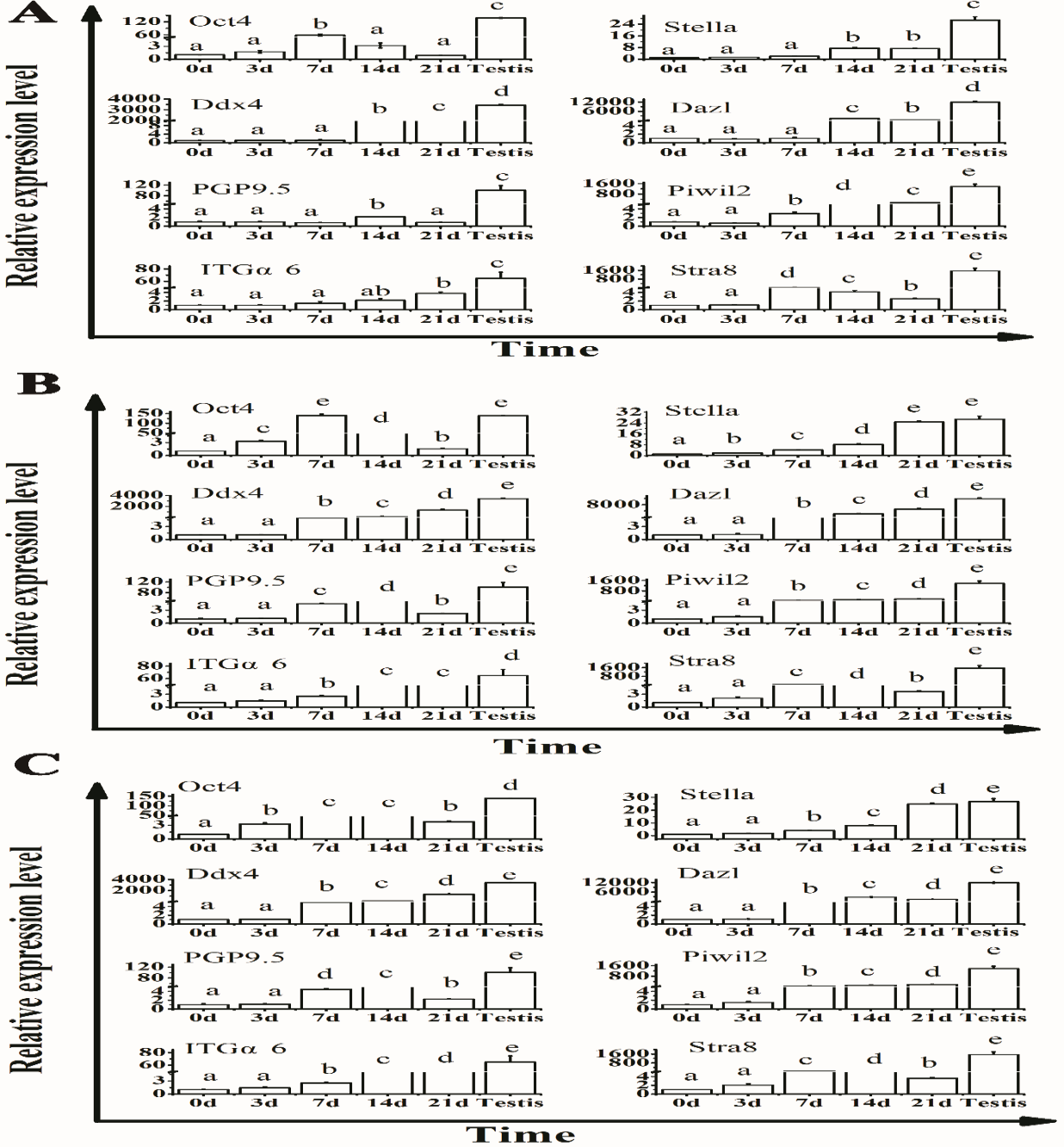
**

**Figure S2 mRNA expression of germ cell (GC)-related markers in induced-ADMSCs on differentiated 0d, 3d, 7d, 14d, 21d by three mode and testis.** The expression of all genes was up-regulated and the trends remain same as in ADMSCs grown under different treatments as a whole, but lower than that of testis. Results are presented as a mean ± SEM of three duplicate runs. Error bars in charts represent the corresponding standard deviations. Y-axis indicated the relative mRNA value normalized to undifferentiated ADMSCs cells mRNA level. (A). Individual induction. (B). Indirect co-culture with TM4 cells. (C) Direct co-culture with TM4 cells. Different letters on the bar graph indicate significant differences between the groups at the 0.05 level (n=3).

**Figure S3**


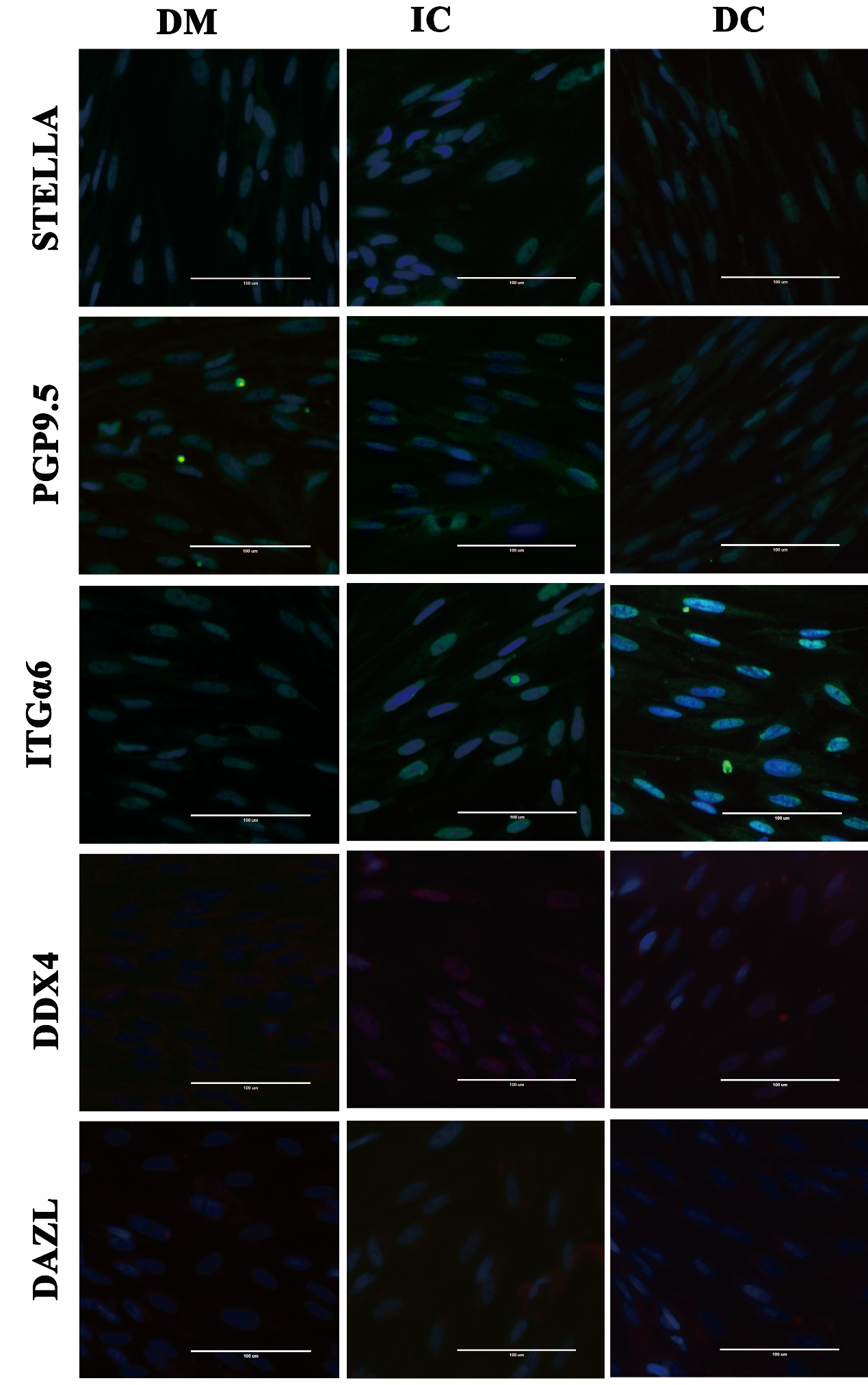


**Figure S3 Protein expression of germ cell (GC)-related markers in induced-ADMSCs on differentiated 21 d by three modes.** DM: Individual induction (left), IC: Indirect co-culture with TM4 cells (center), DC: Direct co-culture with TM4 cells (right). (Scale bar 100μm). Stella (green), DDX4 (red), Dazl (red), PGP9.5 (green) and ITGα6 (green) were observed in ADMSCs from different treatment group.

**Figure S4**

**
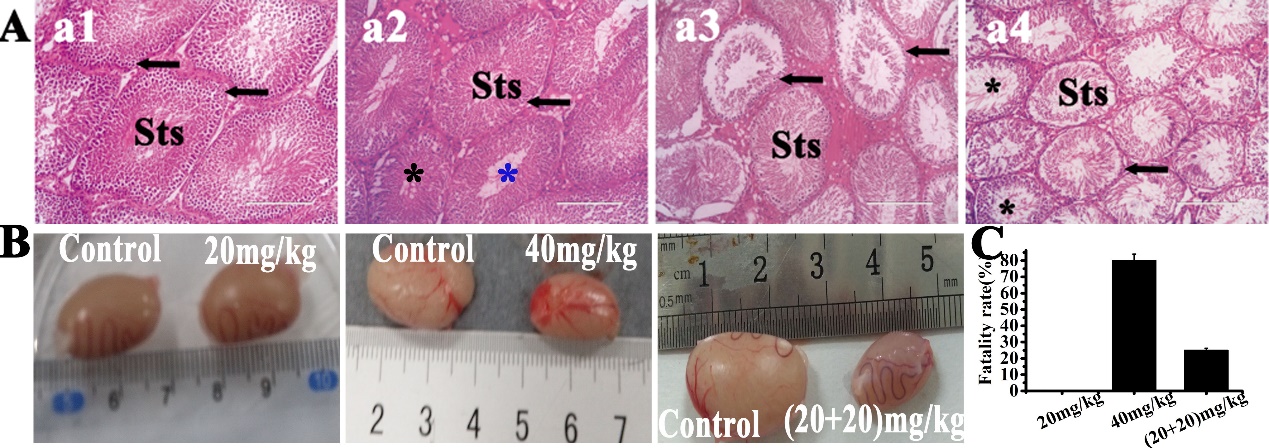
**

**Figure S4 Busulfan induced azoospermatism model preparation after four weeks under different** treatment conditions. Rats were treated with 50% DMSO as control; the rats were treated with either intraperitoneal injection of 20 mg/kg (20 mg/kg) or 40 mg/kg (40 mg/kg) busulfan once, or 20 mg/kg busulfan, twice with 14 days of interval ((20+20) mg/kg). (A). Hematoxylin and eosin-stained sections of the testis. (a1) Control rat testis demonstrated normal morphology. The seminiferous tubules (Sts) are densely packed, in which Sertoli cells (curved arrow) and spermatogenic cells are arranged regularly**.** (a2) 20 mg/kg treatment group rat testis shown few distorted Sts, nevertheless, the Sts without sperm (blue asterisk) and sparse spermatogenic cells (black asterisk) can be easily observed. (a3) 40 mg/kg treatment group rat testis shown most of distorted Sts, spermatogenic cells are widely separated, indicating degeneration. (a4) (20+20) mg/kg treatment group rat testis shown most of distorted Sts, most of them containing no sperm and spermatogenic cells are widely separated, indicating degeneration, but, loss of spermatogenic cells are observed in some Sts (black asterisk). (Scale bar 200μm). (B). Comparison of testicular size, Compared with the control groups, testicles size of 40 mg/kg group and (20+20) mg/kg group is much smaller, while there is no difference of 20 mg/kg group. (C) Fatality rate analysis. 40 mg/kg Group has the highest fatality rate (80%), followed by (20+20) mg/kg group (25%) and there is no rat dead in 20 mg/kg group.

**Figure S5**

**
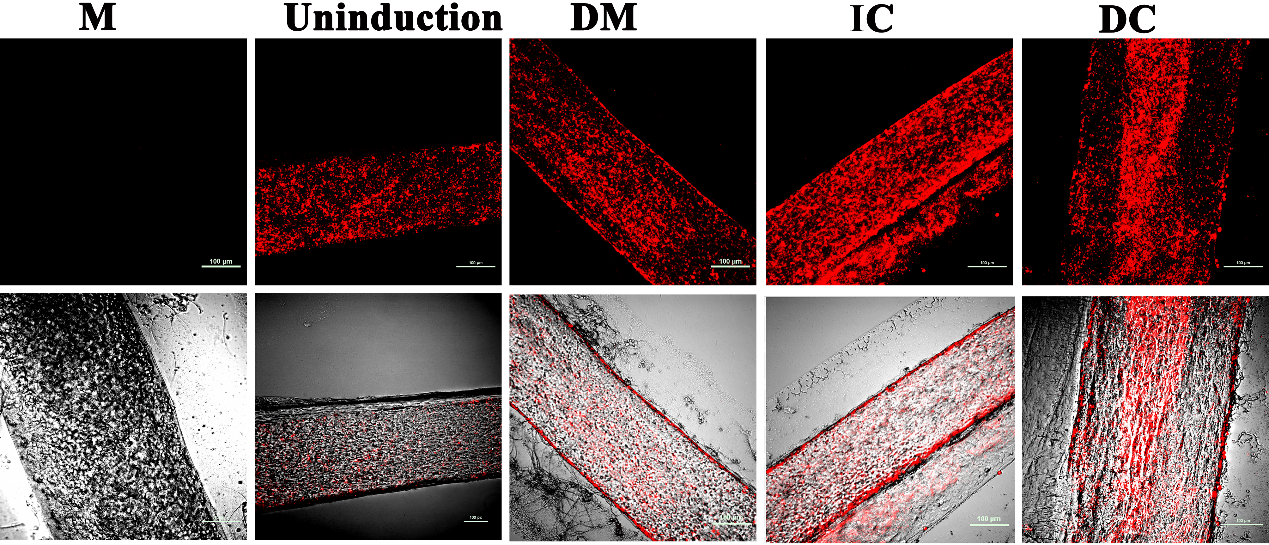
**

**Figure S5 Survival of transplanted-ADMSCs in seminiferous tubules analysis.** PKH26 labeled-ADMSCs from *in vitro* treatment group can survive in the seminiferous tubules of recipient testis 2 months after transplantation. (Scale bar 100μm).

**Figure S6**

**
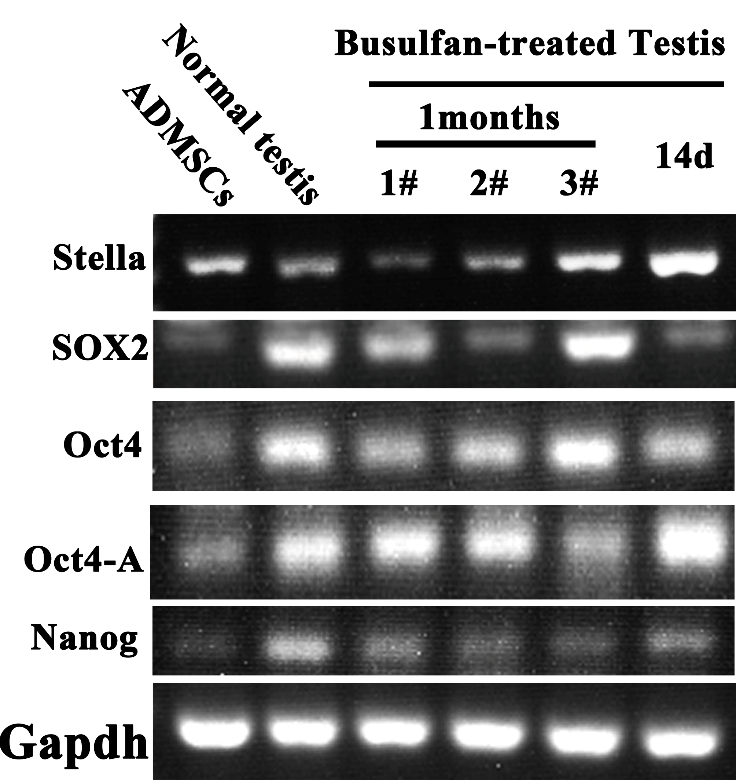
**

**Figure S6** Comparing the expression of multipotential and primary germ cell related markers such as OCT-4, OCT-4A, Sox2, Stella and Nanog in rat testis with or without busulfan treatment by RT-PCR.

**Figure S7**

**
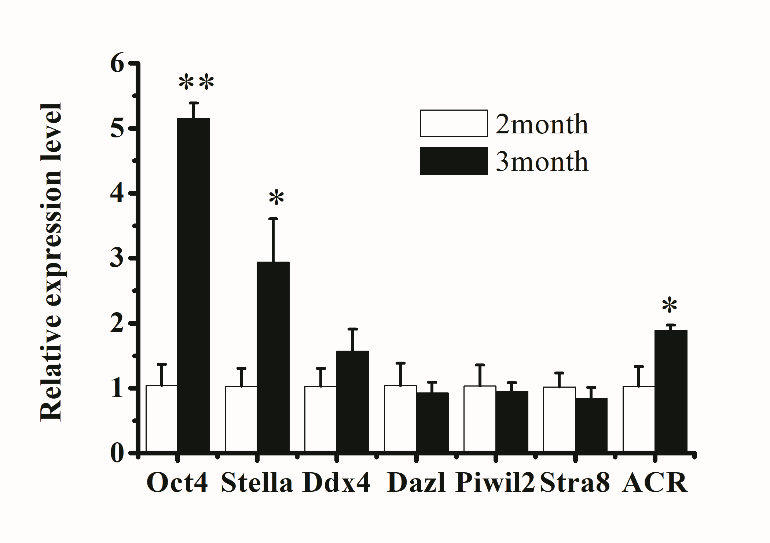
**

**Figure S7 The mRNA expression of germ cell (GC)-related markers in rat testis after busulfan-induced 2 and 3 months**. Oct4, Stella and ACR mRNA expression is significantly up-regulated in rat testes of busulfan-treated for 3 months (black bar) than 2 months (white bar), indicating the self-repairing of spermatogenesis in rat testicular after busulfan treatment. Bar graphs represent mean±SD (n=3 per group).*p<0.05, **p<0.01 vs control.

**Figure S8**

**
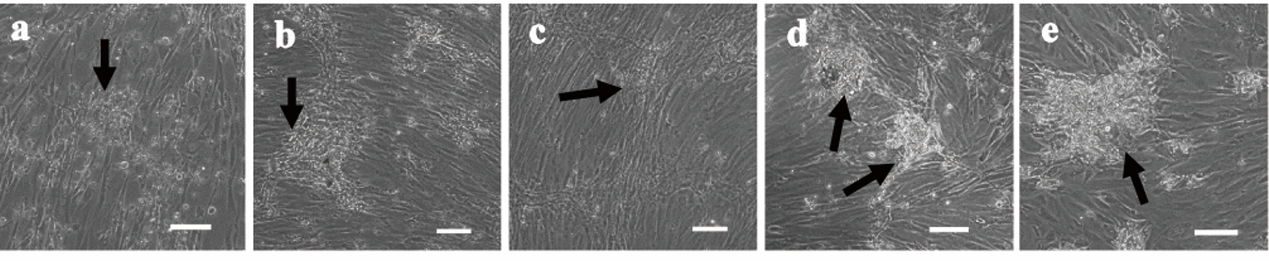
**

**Figure S8 Morphology of ADMSCs *in vitro* treated by Mitomycin C inactivated TM4 cell, TM4 cell-Conditioned Medium (TCCM), RA and T on 21d.** ADMSCs after treated by mitomycin C inactivated TM4 cell, TCCM, RA and T generated colonies with germ cells (GCs) features (black arrow).**(a). Treatment with RA and T (DM). (b).** Combination of RA and T treatment with indirectly co-cultured with TM4 cell(IC)**. (c).** Indirect co-culture with mitomycin C inactivated TM4 cell (TM4 cell). **(d)** Treatment with TCCM, RA and T D M: treatment with RA and T (TCCM+RA+T). **(e)** Treatment with TCCM (TCCM). (Scale bar 100μm).

**Figure S9**

**
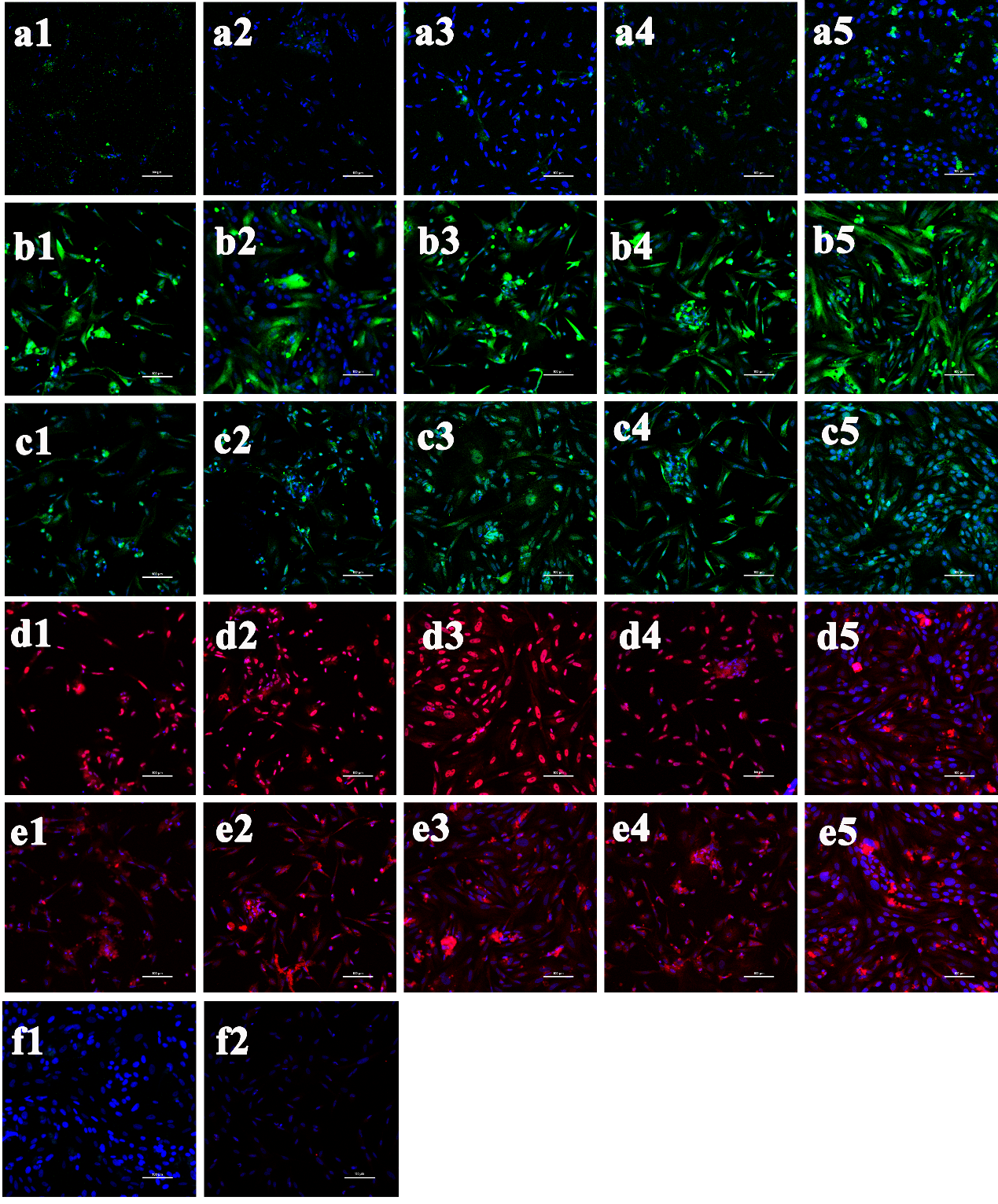
**

**Figure S9 Protein expression of germ cell (GC)-related markers in ADMSCs treated with Mitomycin C inactivated TM4 cell, TM4 cell-conditioned Medium (TCCM), RA and T on 21d by immunostaining analysis.** ADMSCs after treated by mitomycin C inactivated TM4 cell, TCCM, RA and T expressed the protein of GCs-related markers, such as Stella, PGP9.5, ITGα6, DDX4 and Dazl. **(a1-e1)**. Treatment with RA and T (DM). **(b2-e2).** Indirect co-culturing with mitomycin C inactivated TM4 cell (TM4 cell). **(c3-e3)** Treatment with TCCM (TCCM). **(d4-e4)** Combination of RA and T treatment with indirectly co-culturing with TM4 cell (IC). **(d5-e5).** Treatment with TCCM, RA and T D. Stella (a1-a5), PGP9.5 (b1-b5) and ITGα6 (c1-c5) were assessed by immunohistochemistry (IHC) analysis, PBS as a negative control (f1)**.** DDX4 (d1-d5) and Dazl (e1-e5) were assessed by immunofluorescence staining analysis, Cy3-conjugated rabbit IgG cultured under the same medium was used as an isotype control(f2). (Scale bar 100μm).

**Figure S10**

**
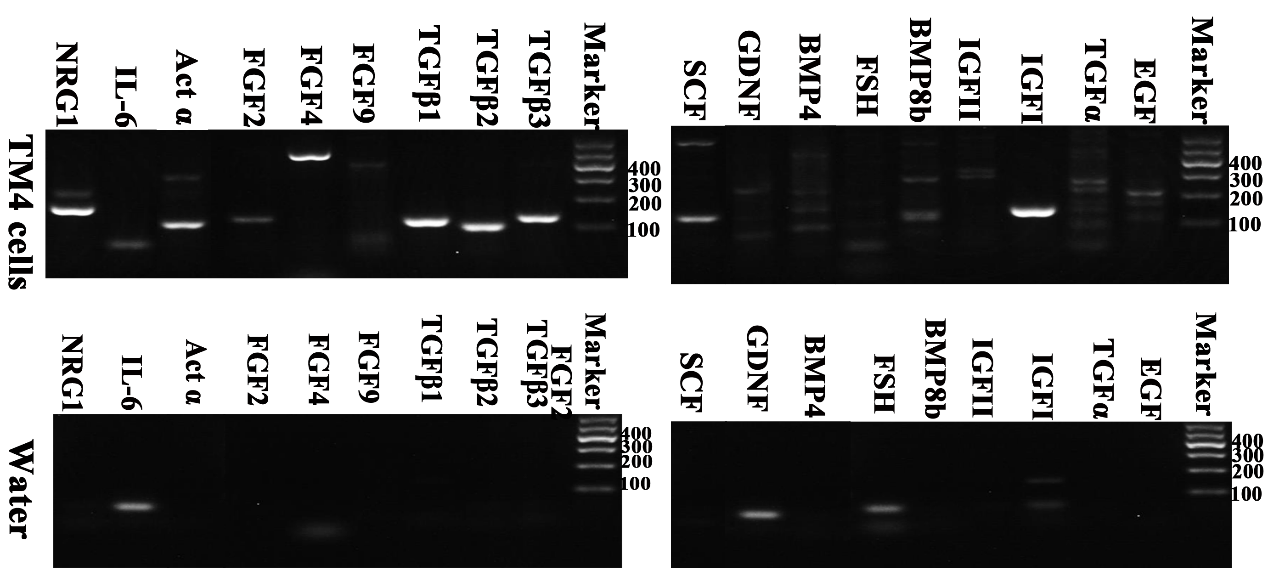
**

**Figure S10** mRNA expression of cytokines in TM4 cells by RT-PCR. PCR template from mRNA isolated from TM4 cells (TM4 cells) (above), PCR template is high temperature sterilization deionized water used as negative control (Water) (below). The results shown that TM4 cells expressed a large number of FGF2, IGFI, NRG1, SCF, Actα, TGFβ1, TGFβ2 and TGFβ3 genes, a small amount of BMP8b, BMP4 and EGF genes. However, hardly express GDNF, FSH, IGFII, TGFα genes.

**Figure S11**

**
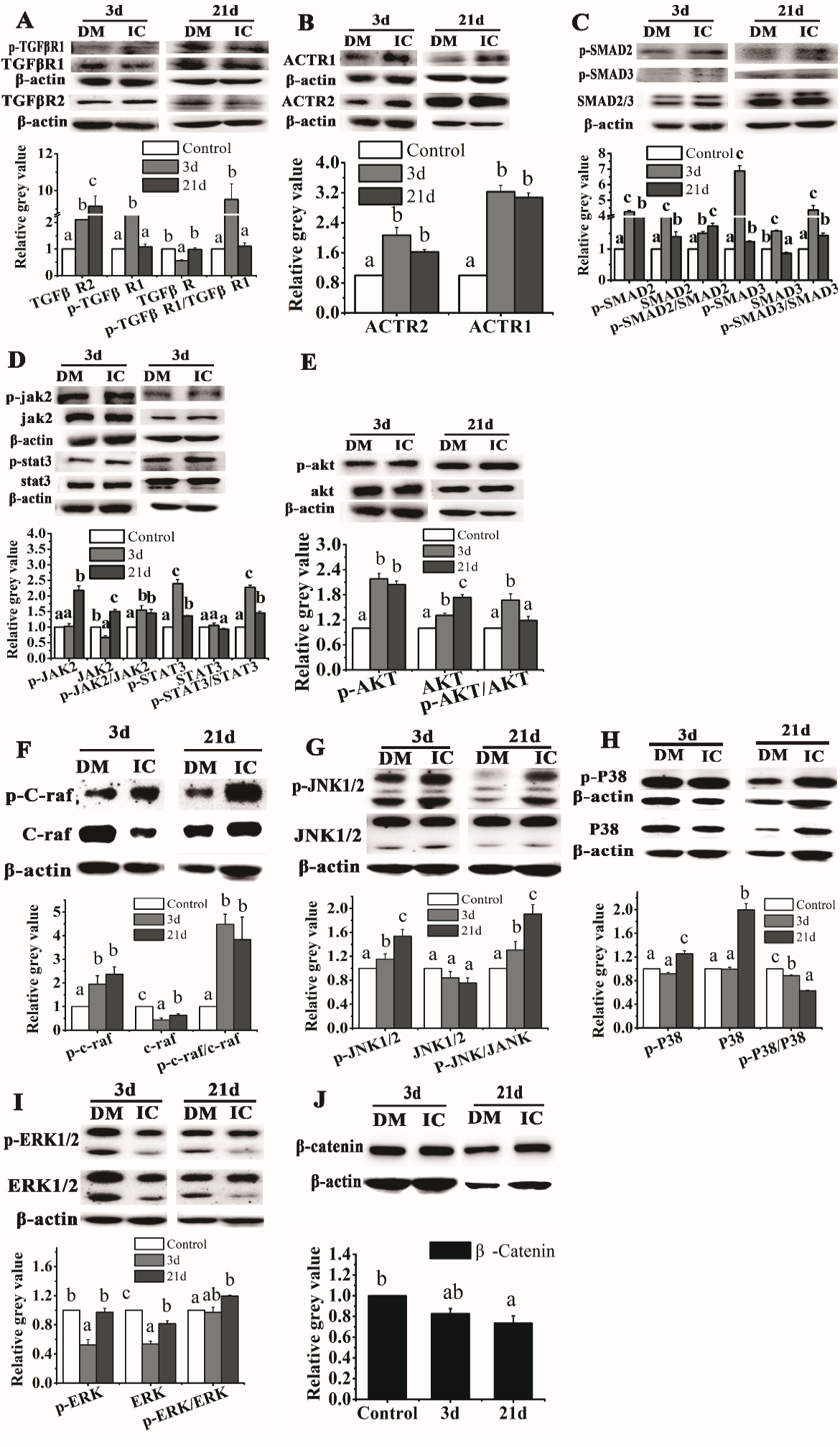
**

**Figure S11** Relative protein expression of key factors of TGFβ/Smad2/3, JAK2/STAT3, AKT, Wnt/β-catenin, mitogen activated protein kinases (MAPKs), ERK1/2, p38 and JNK signaling pathway on differentiated 3d and 21d under DM and IC group culture condition cells by western blot analysis. ADMSCs cultured in DM group condition as control. DM: treatment with RA and T (white bar); IC: Combination of RA and T treatment with indirectly co-culturing with TM4 cell, on differentiated 3d (gray bar), on differentiated 21d (dark gray bar). Quantitative analysis was performed by image-pro plus software, Bar graphs represent mean±SD (n=3 per group), Y-axis indicates the relative grey value normalized to DM group grey value, different letters on the bar graph indicate significant differences between the groups at the 0.05 level (n=3). **(A-C)** Indirectly co-cultured with TM4 cell activated TGFβ/Smad2/3 signaling pathway. **(D)** Indirectly co-cultured with TM4 cell activated JAK2/STAT3 signaling pathway. **(E)** Indirectly co-cultured with TM4 cell activated AKT signaling pathway. **(F)** Indirectly co-cultured with TM4 cell activated MAPK signaling pathway. (G). Indirectly co-cultured with TM4 cell activated JNK signaling pathway. (H). Indirectly co-cultured with TM4 cell inhibited p38 signaling pathway. **(I)** Indirectly co-cultured with TM4 cell have no effect on ERK signaling pathway. **(J)** Indirectly co-cultured with TM4 cell inhibited Wnt/β-catenin signaling pathway. TGFβ/SMAD2/3, JAK2/STAT3 and PI3K/AKT signaling pathways were found to be significantly affected than others. These signaling pathways were further analysis by corresponding signal pathway inhibitors.
